# Circuit Topology Approach for the Comparative Analysis of Intrinsically Disordered Proteins

**DOI:** 10.1021/acs.jcim.3c00391

**Published:** 2023-04-07

**Authors:** Barbara Scalvini, Vahid Sheikhhassani, Nadine van de Brug, Laurens W. H. J. Heling, Jeremy D. Schmit, Alireza Mashaghi

**Affiliations:** † Medical Systems Biophysics and Bioengineering, Leiden Academic Centre for Drug Research, Faculty of Science, Leiden University, Einsteinweg 55, 2333 CC Leiden, The Netherlands; ‡ Centre for Interdisciplinary Genome Research, Faculty of Science, Leiden University, Einsteinweg 55, 2333 CC Leiden, The Netherlands; § Department of Physics, Kansas State University, Manhattan, Kansas 66506, United States

## Abstract

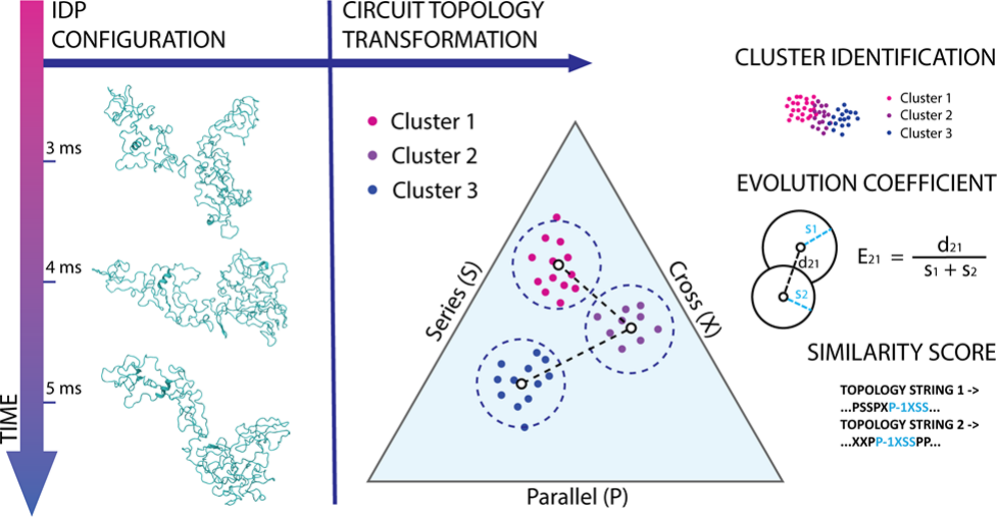

Intrinsically disordered proteins (IDPs) lack a stable native conformation, making it challenging to characterize their structure and dynamics. Key topological motifs with fundamental biological relevance are often hidden in the conformational noise, eluding detection. Here, we develop a circuit topology toolbox to extract conformational patterns, critical contacts, and timescales from simulated dynamics of intrinsically disordered proteins. We follow the dynamics of IDPs by providing a smart low-dimensionality representation of their three-dimensional (3D) configuration in the topology space. Such an approach allows us to quantify topological similarity in dynamic systems, therefore providing a pipeline for structural comparison of IDPs.

## Introduction

Until recent years, the dogma in protein biology entailed that functional proteins or domains have unique and stable three-dimensional (3D) structures. These native configurations can be characterized by their virtually fixed atomic positions and backbone Ramachandran angles, which vary only slightly as a result of thermal fluctuations. However, there exists another class of functional proteins which contain highly dynamic regions or are characterized by the absence of apparent ordered structure under physiological conditions. These proteins have no single, well-defined equilibrium structure but exist as heterogeneous ensembles of conformations that cannot be sufficiently described by a single set of geometric coordinates or backbone Ramachandran angles.^1,2^ These proteins, present in all kingdoms of life, are biologically active and adapt to a highly specific structure upon important functional interactions with biological partners.^3^ They have been called many names,^4^ but are now commonly referred to as intrinsically disordered proteins (IDP) or intrinsically disordered regions (IDR). It is estimated that more than 30% of all proteins in the eukaryotic proteome are either entirely disordered or contain disordered regions of more than 50 consecutive amino acids.^5^ This fraction of the proteome includes crucial proteins involved in essential biological functions, like signaling,^6^ transcriptional control,^7^ and allosteric regulation.^8^ Mutations in these proteins thus might play a role in disease development.^9^ Indeed, IDPs and IDRs are implicated in many pathologies ranging from cancer^10^ and metabolic diseases to neuromuscular disorders^11^ and have been suggested as an attractive target for therapeutic interventions.^12^ For this reason, an understanding of the structure–function relation in these disordered molecules is paramount. The conformational disorder poses serious challenges for experimental and computational analysis of IDP/IDR conformations and interactions and, to date, even the most state-of-the-art machine learning approaches have been unable to successfully elucidate the native structures of disordered proteins and regions.^13^ Despite these challenges, modeling^14,15^ and experimental^16,17^ investigations have led to important insights into the functional dynamics of these intrinsically disordered proteins (IDPs).^18,19^

**Figure 1:**
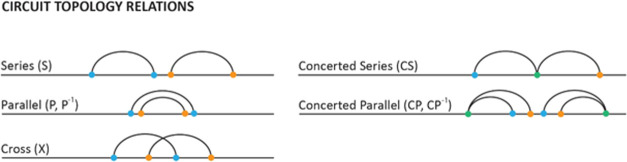
Circuit topology relations: each pair of contacts can be characterized by one of three relations: series, parallel, and cross. The topological relation between pairs of contacts is assigned based on the order in which contact sites (residues) appear along the sequence. Sometimes one contact site is shared between contacts (green dots in the panel). In this case, we talk about concerted relations, which are a subset of either S or P relations.

What hampers our understanding of these proteins is the lack of a proper description of the dynamics that captures topological motifs, hidden within the conformational noise. Furthermore, there is a need for a “reaction coordinate” to map the interconversion of potential motifs. Topology is a mathematical framework that is designed to detect such shape invariants in geometric ensembles. Recently, topology of unknotted protein chains has been defined based on the arrangement of loops or the associated intrachain contacts. This approach, called circuit topology (CT),^20−22^ has been applied to stable folded proteins for various applications,^23,24^ and has proven to be effective for modeling polymer folding reactions.^25^ CT is a very simple yet effective framework for the characterization of the arrangement of interchain (residue–residue) contacts in a folded molecule. The core idea is that the arrangement of any pair of contact belongs to either one of three topological relations: series (S), parallel (P), and cross (X) (Figure 1). The assignment of topological relations relies on the numbering and positioning of contact sites along the chain sequence. Contacts belonging to the S class are spatially “noninteracting”: their contact sites appear serially along the chain, and the contacts do not intersect. On the other hand, a contact which is fully encompassed by another contact is said to be in P relation with the latter. Finally, contacts in X relation “interact” spatially, but one is not fully enveloped by the other. These three relations characterize all possible contact arrangements within a chain. It is possible for two of these relations, series and parallel, to share one of the contact sites between the contact pair (Figure 1). In this case, we call this subclass concerted relations, resulting in concerted parallel (CP) and concerted series (CS).

The CT approach has not yet been applied to disordered proteins. Since intrinsic disorder does not mean random, we believe such a framework could capture conserved features in the wide topological evolutions of such systems. Moreover, we suggest this method could be able to detect topological similarity between IDPs with similar function, providing a new metric for the quantification of structural similarity suitable for IDPs and proteins with a stable 3D structure alike. Here, we coupled circuit topology and molecular dynamics (MD) simulations for IDP analysis and applied it to the disordered N-terminal transactivation domains (NTDs) of three proteins from the family of nuclear hormone receptors (NHR), namely, human androgen receptor (AR), glucocorticoid receptor (GR), and estrogen receptors (ER). We mapped the folding dynamics of the NTD domains onto the topological space, providing reaction coordinates to finally visualize the intrinsically disordered conformational dynamics. We performed a comparative analysis of these disordered receptor domains, using the disordered γ-synuclein (residues 1–114) and a few well-folded proteins as references. We prove how it is possible to find common traits characterizing such conformational evolution, while also identifying differential patterns of behavior among our protein dataset, ranging from the extent and dynamics of topological evolution, as well as the topological content itself. Modeling intrinsically disordered proteins poses significant challenges due to the limited sampling capabilities of their flat energy landscape.^26^ Here, we do not aim at offering a solution to such challenges but rather present a smart data representation for the topological characterization and comparison of IDPs.

## Results

### Basic One- (1D) and Three-Dimensional (3D) Comparative Analysis of NHR Dynamics

**Figure 2:**
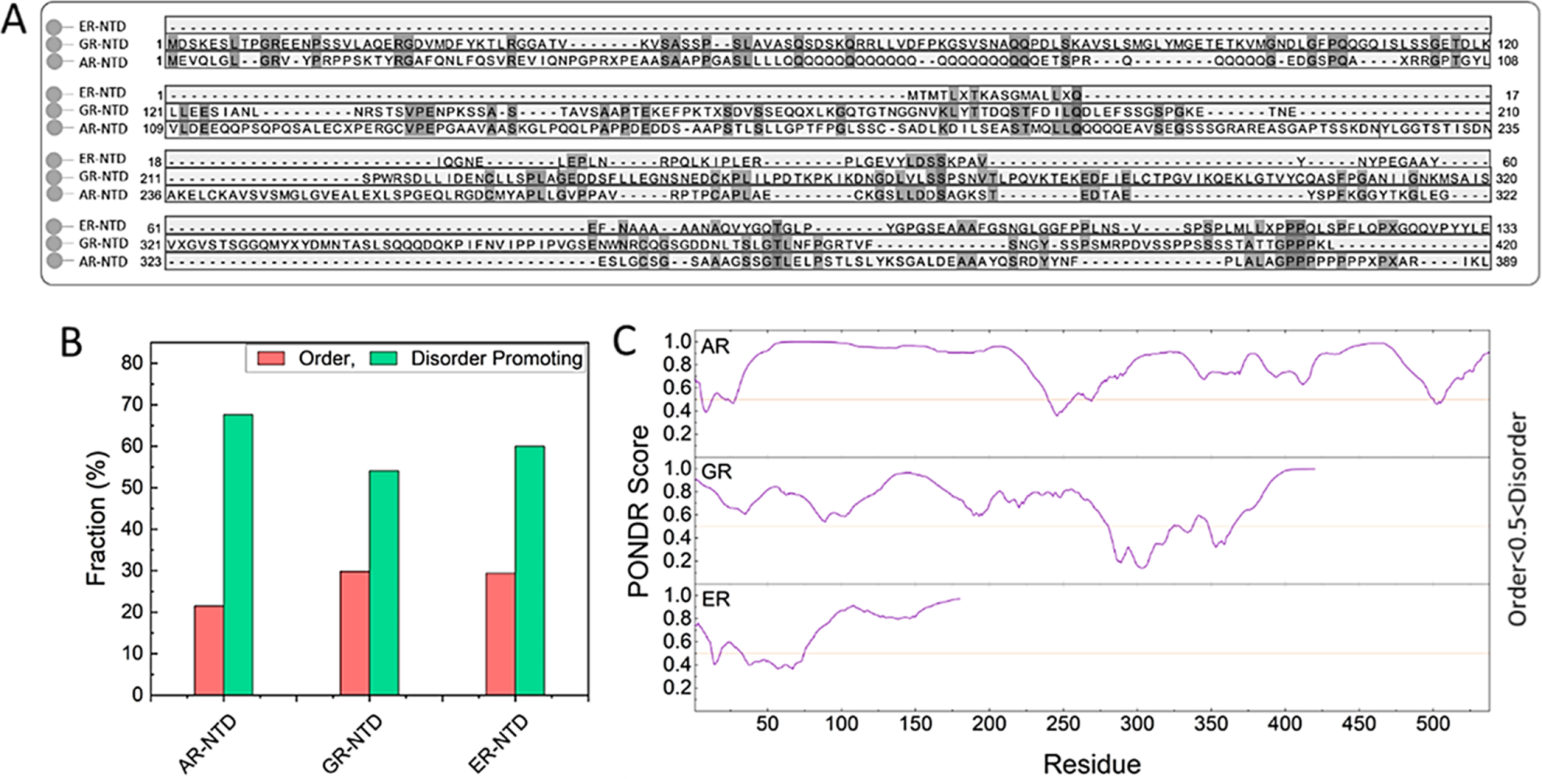
Sequence analysis of NTDs. (A) Multiple sequence alignment of the AR, GR, and ER NTDs. (B) Fraction of order and disorder-promoting residues calculated based on the amino acid content of the chains. (C) Structural disorder analysis of AR-NTD obtained from PONDR VSL2.

As a case study, we focus on a comparative analysis of NTD regions of three hormone receptors, including AR (residues 1–538), GR (residues 1 to 420), and ER (residues 1–180). We first looked at the amino acid composition of the chains and performed multiple sequence alignment (MSA) and PONDR analysis.^27^ MSA showed nonsignificant similarity between the NTDs, but by comparing the sequences pairwise, we saw more matching residues between ER, GR, and the C-terminal half of AR (Figure 2A). Disorder prediction data produced by PONDR analysis reveal that all three chains are highly disordered. To further understand the dynamic nature of these chains, we calculated the order (OPR) and disorder-promoting residues (DPR) content. For all three NTDs, we found a high DPR content (Figure 2B,C). As a comparative analysis, the same parameters were calculated for intrinsically disordered γ-synuclein (SNCG), which showed 64 and 24% disorder- and order-promoting amino acids content, respectively, and an average PONDR score of 0.83 ± 0.10.

**Figure 3:**
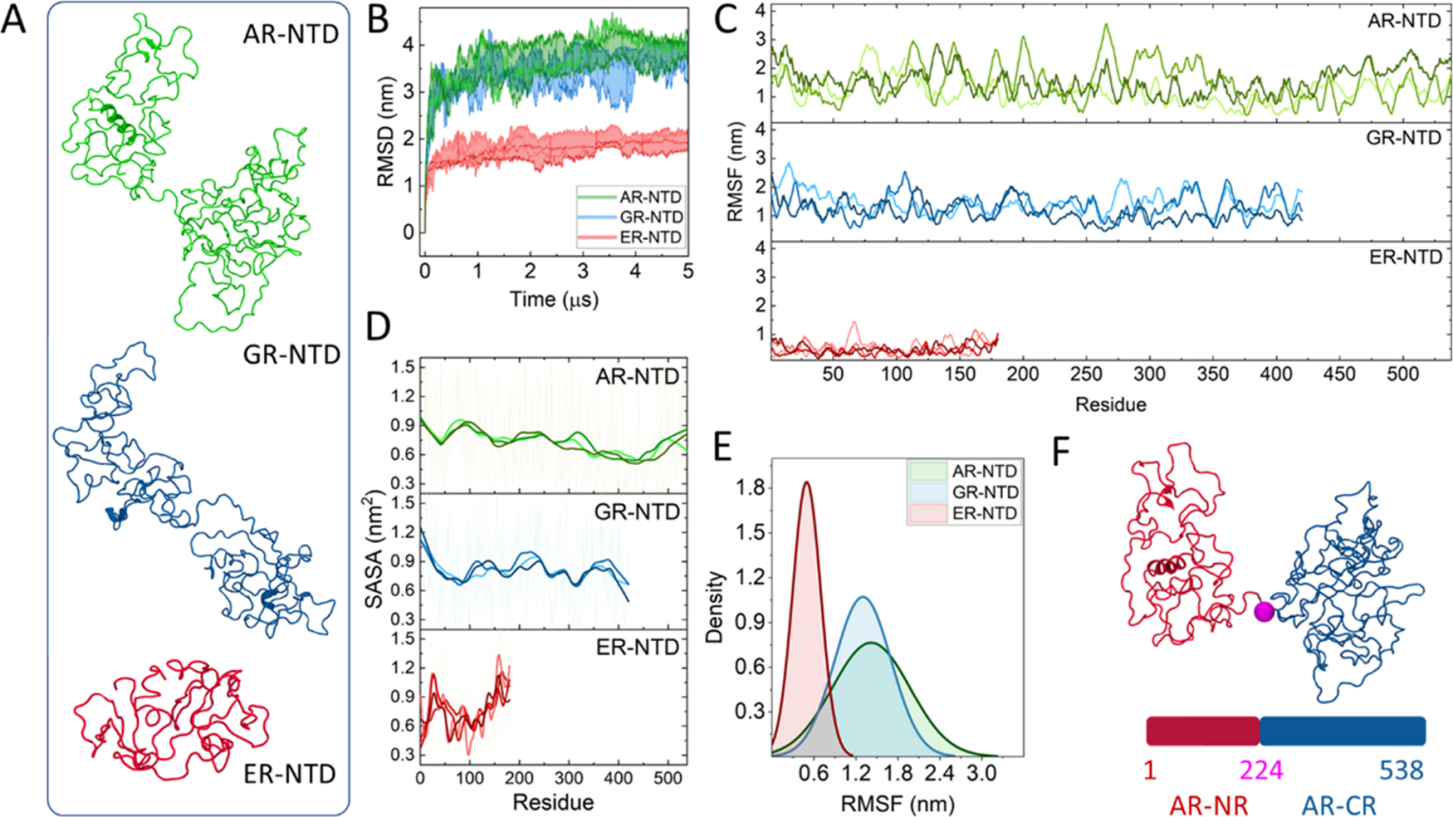
Molecular dynamics simulation of NTDs. (A) Three representative conformations from the last 10 ns of each replicate of the MD simulations. (B) Time evolution of the root means square deviation (RMSD) of three runs of each NTD. All three NTDs show a dramatic deviation from the initial structure (reference frame) during the first 2 μs of the simulations. (C) Average root mean square fluctuations of AR-NTD were calculated per residue over the last 3 μs of the simulation. (D) The solvent-accessible surface area (SASA) was calculated for each residue during the last 3 μs of the simulation. (E) Distribution of RMSF values calculated per residue from the last 3 μs of the simulations. (F) Cartoon representation of the AR-NTD. Two disjoint regions are formed within 2 μs of simulations: an extended N-terminal subregion (NR, residues 1–224) colored in red and a C-terminal subregion (CR, residues 225–538) colored in blue. Bead representation of residue 224 is colored in pink.

Next, we modeled the dynamics of these three protein domains in an aqueous solution with physiological salt concentration, to develop reasonable toy models for the proof-of-concept topological analysis. We note that modeling large disordered protein chains is challenging due to the limited accuracy of the force fields used to model interactions and the need for adequate sampling of the large conformational space of the solvated chain. Here, we took a practical approach and employed our recently developed and experimentally validated protocol for AR NTD analysis^28^ on GR and ER protein chains. The initial structures, for all three NTDs, were built using the I-TASSER^29^ server and choosing the best-ranked model. The model was superior to conformations predicted by the AlphaFold based on confidence measures. After minimization and relaxation, we performed molecular dynamics simulations of the full-length NTD structures (see the Methods section for details). Visual examination of the trajectory and root mean square deviation (RMSD) plots show that the initial conformations have undergone an extensive structural change (Figure 3A,B). We repeated the MD simulation three times for each NHR using different initial velocities to ensure we had a sufficient sampling of the configuration space for the purpose of this study. Importantly, all three independent runs of all three NHRs consistently resulted in the emergence of compactness in the chain within 2 μs of simulations. Interestingly, among the three, AR formed two disjoint regions in Figure 3D within 2 μs of simulations: an extended N-terminal subregion (AR NR, residues 1–224), and a C-terminal subregion (AR CR, residues 225–538), as reported extensively in our previous study^30^ (Figure 3F). In contrast, ER-NTD stayed as a whole globularly shaped conformation during the three runs of the simulations (Figure 3A). GR formed a few identifiable globular regions, which were interconnected with each other. Despite the overall shape taken by the chains, all three showed a high level of disorder and structural dynamics (Figure 3C–E).

After the initial folding phase, we monitored the dynamics for an additional 3 μs and computed root mean square fluctuations (RMSF) to quantify the fluctuations of the chain. Interestingly, RMSF analysis led to the largest values in AR and significantly smaller in ER. It is worth mentioning that these values were significantly larger in comparison to the folded NHR-LBD, even for ER-NTD, with lowest RMSF values among the three NHRs (Figure 3C). Further analysis of the RMSF profiles (Figure 3E) revealed that in ER-NTD the fluctuations were more uniformly distributed within the chain, and distribution analysis showed a sharp peak at 0.5 nm. However, GR- and AR-NTD both had wide distributions with mean values at 1.5 nm and 1.3 nm, respectively.

Due to the highly dynamic nature of the chains, it was expected to see a large part of the chains be exposed to the solvent. In order to quantify that, we calculated solvent-accessible surface area (SASA) of the polypeptide chains. SASA analysis revealed that all three NTDs are highly solvent-accessible (Figure 3D). Among them, ER-NTD showed the widest range of exposure from 0.35 nm^2^ (residues buried inside a compact region) to 1.3 nm^2^, (residues are fully accessible to the solvent molecules).

Formation of the collapsed region(s) within the chain was the common behavior of the NTDs we observed in our simulations. In order to quantify the degree of compactness, we calculated the radius of gyration (RG) values over the last 3 μs of the simulations, separately for NR and CR regions of AR and full-length ER-NTD. Comparing the radii of gyration of CR and NR regions in AR, one can clearly see that the CR region is significantly more compact than the NR region (Figure S1) and both are less compact in comparison to the full-length ER-NTD. Note that all RG values are normalized to the size (Flory radius with ν = 1/3) of the corresponding region(s).

Disorder prediction data produced by PONDR analysis agrees with the solvent accessibility and RMSF profiles of three NTDs: with the central region within AR CR and GR having less disorder than the rest of the chain (Figure 2C) and high disorder score predicted for the C-terminal half the ER-NTD. For ER-NTD, a high and low disorder score predicted for the N-terminal half of the chain is nicely matched with the SASA profile of residue 20–80. Furthermore, we clearly saw that the OPR content of the NR region was significantly less than the CR (18–23% of OPR content compared to 64–72% of DPR content, Figure S2). This is in an agreement with the PONDR score, SASA, and RG values calculated for CR and NR regions.

## Multi-Timescale Topological Analysis of IDP Conformational Evolution

**Figure 4:**
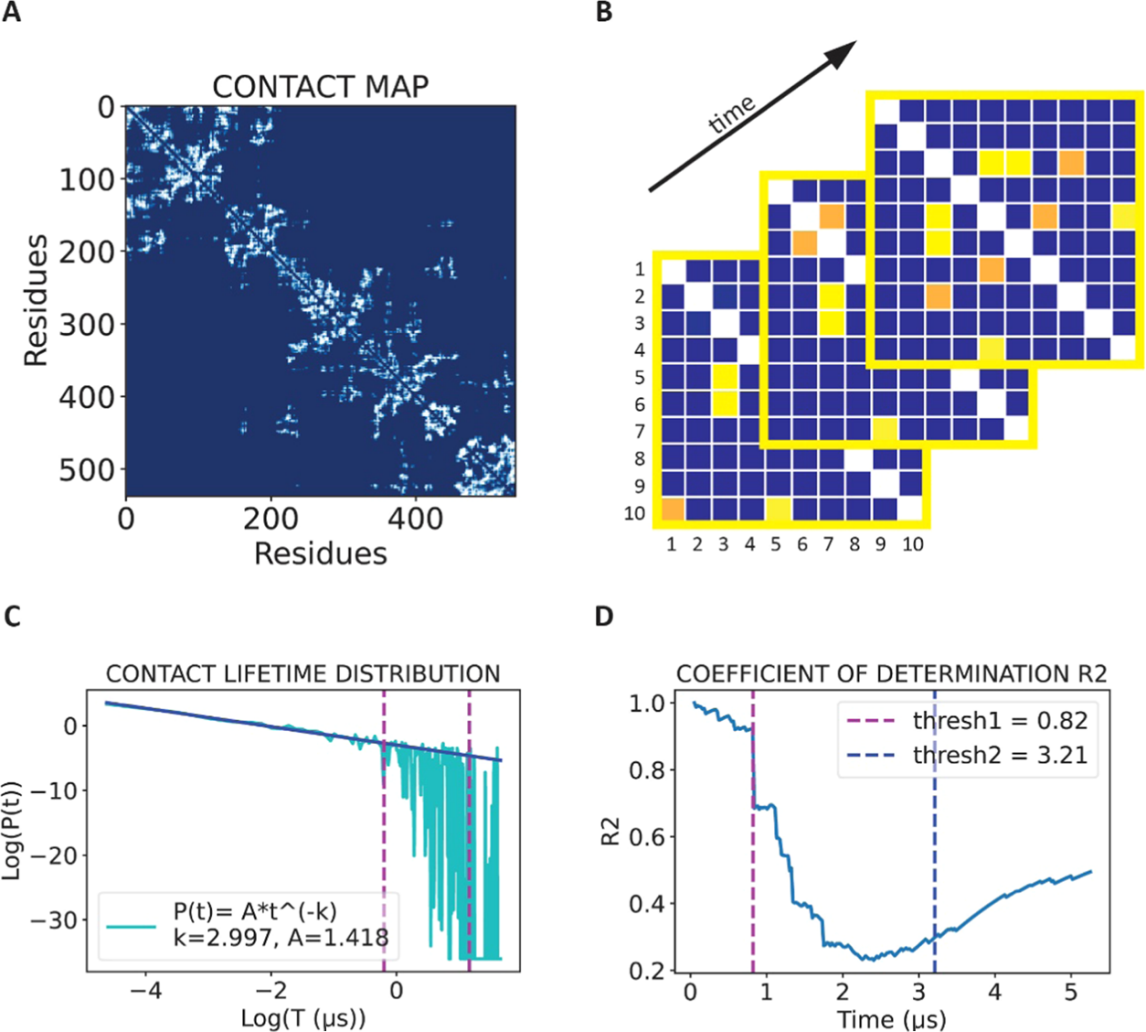
Adherence to the power law distribution can help us distinguish between short- and long-lived contacts. (A) Cumulative contact map of AR NTD, MD run 1. The subdivision into two subregions (NR and CR) can be seen in the contact arrangement patterns. (B) Graphics representing three contact maps, corresponding to three different time frames of a hypothetical IDP. Contacts represented in yellow are present in all three frames because of their long lifetime. Contacts represented in orange are on the other hand short-lived, and they disappear in subsequent time frames because they are short-lived. The presence of specific contacts over different time frames is detected in order to build the contact lifetime distribution. (C) Contact lifetime distribution, and power law fit for AR-NTD, MD run 1. The fit was performed exclusively over short life contacts, and then extrapolated over the whole range, for visualization purposes. (D) Coefficient of determination *R*^2^, used to evaluate the goodness of the power law fit performed over subsequent chunks of the contact lifetime distribution. After roughly 0.5–1 μs, *R*^2^ plummets. We picked this threshold in order to distinguish between short-lived contacts and contacts with a longer life, which make up only a smaller portion of the total number of contacts.

The dynamic behavior of IDPs can hardly be characterized by focusing on a single timescale.^31,32^ Here, we develop a multi-timescale topological analysis, and we prove that different dynamic modes of IDP conformational search can present different topological characteristics. The timescale analysis reported here is a generalization of the procedure applied in our previous study^30^ to the AR-NTD. To this end we will be focusing on the characteristic time frame for contact dynamics, that is to say, contact formation and rupture. The rationale behind this choice is that interchain-interaction topology has been proven to be an efficient way to characterize IDP configurational search and functional similarity.^33^ Our MD simulations provide us with very detailed information about atom coordinates and residue–residue contacts, as well as their temporal evolution (with a resolution of 5 ns). We define contacts between residues when those residues lie within a distance in the 3D space that is less than a specified cutoff (4.5 Å for the purpose of this study). Figure 4A displays a residue–residue contact map for AR, MD run 1. Here, all contacts formed during the simulation are displayed, making this a cumulative contact map for all of the temporal evolution. It is interesting to see how the separation between the N- and C-terminal regions of AR-NTD is also visible from the map, highlighting a very clear boundary for the spatial range of contact formation. For this reason, as well as the different physical and geometrical characteristics of CR and NR highlighted in the previous section, we decided to treat these two regions separately for topological analysis. The MD frames give us access not only to the spatial but also temporal range of contacts, allowing us to measure the duration of contacts formed by each residue pair, as shown in the schematics presented in Figure 4B; different time frames present different configurations. Some contacts survive for multiple time frames (contacts depicted in yellow), while others will be more fleeting connections, breaking in the span of one (or few) MD frames (contacts depicted in orange). We can compute the maximum lifetime of each individual contact (hereafter referred to as lifetime) and build a distribution of contact lifetimes. The log–log plot of such a distribution (Figure 4C, Figure S3) presents us with the opportunity of describing the phenomenon of contact formation as a power law distribution, as many other processes in biology, such as scale-free networks.^34^ However tempting, this theoretical approximation may sound, identifying power law distributions on empirical data presents various challenges, mostly given by the large fluctuations characterizing the right tail of the distribution, the one characterized by large but rare events.^35^ For this reason, we decided to tread carefully and define clear boundaries for the validity of the law by quantifying the agreement with the data by use of the determination coefficient *R*^2^ (Figure 4D). We will also use this agreement to disentangle the role of high-frequency contact formation and breaking from that of longer-lived connections, which might impact the configurational evolution in a meaningful way, steering toward a specific local minimum in the topological space. In order to do so, we fit the logarithm of the contact lifetime distribution by progressively larger segments (with increments of 5 ns, which is as low as our resolution allows us to reach). For each segment, we calculate the coefficient of determination *R*^2^. Plotting the result of this calculation versus time yields trends such as that depicted in Figure 4D, for all proteins (see Figure S4): we observe a good agreement between the law and the data for very short time frames (generally around 1 μs). This range is also where the majority of contact lifetimes lie. From now on, we shall refer to the contacts within this range as short life contacts. Afterward, we observe a drop in values of *R*^2^, reflective of a lack of statistics in the distribution. We call this longer-lived connections middle life contacts. After roughly 3 μs, we start observing a mild increase in *R*^2^, but this increase is an artifact of the noise in the distribution. Long life contacts that live in this range have lifetimes comparable to the total duration of the MD simulation, and we are thus unable to observe their full dynamic evolution.

**Figure 5:**
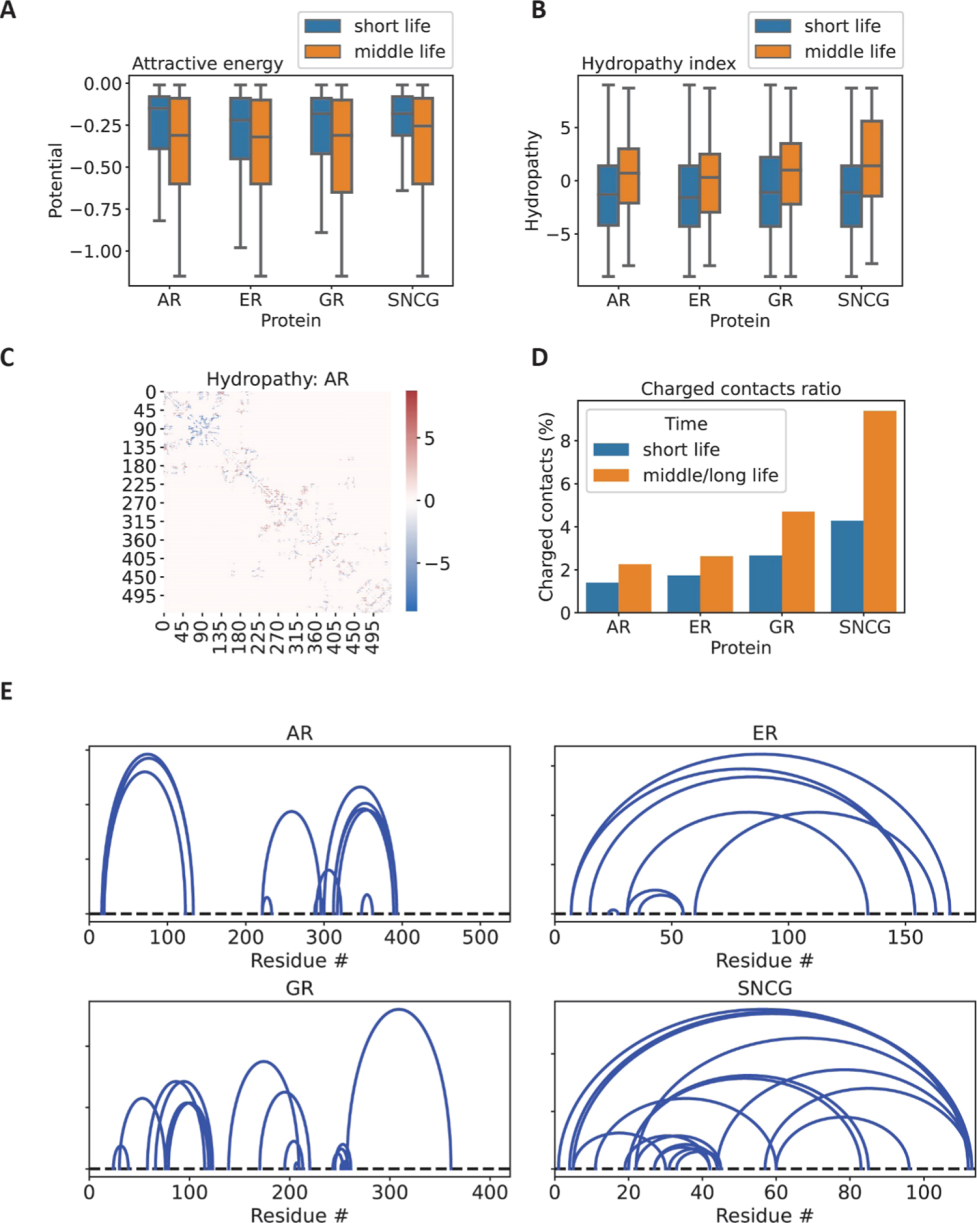
Population of longer-lived contacts is statistically more hydrophobic, has higher attractive energy, and presents a higher ratio of charged contacts than its shorter-lived counterpart. (A) Boxplot of the statistical potential^36^ of short and middle life attractive contacts, for all proteins involved in the study. The two distributions are statistically different, yielding a *p* value <0.05 for all 20 extractions of randomly sampled subpopulations of 300 data points from the two groups. (B) Boxplot of the hydropathy index for short and middle life contacts, for all proteins included in the study. The two distributions are statistically different, yielding a *p* value <0.05 for all 20 extractions of randomly sampled subpopulations of 300 data points from the two groups. (C) Cumulative heatmap of all contacts formed after the first 2 μs of simulation in AR (MD run 1). The coloring is given by the sum of the hydropathy index of the two residues involved in the contact. Positive indexes indicate overall hydrophobic properties in the protein region. (D) Ratio charged versus total number of contacts  for short and middle/long life, for each protein. (E) Circuit diagram of middle/long life charged contacts for each protein included in the study. Data from all three runs are included in each figure.

It is challenging to provide a full biophysical characterization of the nature of these contacts, and thus explain the shape of the lifetime distribution. However, we can rely on statistical indicators to explore the different properties of short, middle, and long life contacts. It is intuitive to assume that longer-lived contacts might have higher contact energies. By exploiting the statistical potential as expressed by Thomas and Dill,^36^ we can assign an energy value to each residue–residue contact. We observe thus that indeed middle life contacts have statistically higher absolute energy values (more negative), when it comes to attractive contacts, for all proteins in the study (Figure 5A). We can go beyond energy considerations and have a look at the chemical nature of the residues involved in these contacts. A simple and useful parameter is the hydropathy index of a residue, a score indicating the hydrophobic/hydrophilic properties of its side chain.^37^ In this instance, we assign a hydropathy score to a contact obtained by summing the hydropathic index of the two residues involved in its formation: the larger the hydropathy index, the higher the hydrophobicity of the amino acids. Applying this procedure to short and middle life contacts reveals that the latter display consistently a higher hydrophobicity than the former (Figure 5B). This crucial information suggests that middle life contacts are those that are more likely to belong to a semistable collapsed structure, as their hydrophobic nature will tend toward shielding the sidechains from the aqueous environment. This simple procedure can also be applied locally, by plotting the hydropathy score over the contact map (Figure 5C). This visualization can interestingly highlight regions in the protein more or less prone to structure formation. In this case, it is clear to see how AR NR has more marked hydrophilic properties than AR CR, which is compatible with the structural properties of the two regions we identified previously.^30^

Both hydrophobic and charged residues are thought to play a role in stabilizing distant parts of primary structures in proteins.^38^ We can identify those contacts that are formed by opposite charge residues (negatively charged–positively charged residues) and what is the lifetime and spatial distributions of such contacts. It is possible to define a ratio between the number of charged contacts and the total number of residue–residue contact combinations for a certain lifetime, . We observe that taken together, middle and long life regimes present a higher charged contact ratio with respect to short life, in all proteins present in the study (Figure 5D). In this case, it was necessary to consider middle and long life regimes as one group, in order to increase statistics: these two groups are composed of a small number of contacts, of which charged contacts are an even smaller subgroup. However, this relative sparsity of information allows us to visualize all such longer-lived charged contacts in one comprehensive circuit diagram (Figure 5E). Circuit diagrams allow us to visualize the topological arrangement between a set of contacts, as well as the residues involved. Topological circuits are a useful tool to interpret such a diagram:^23,39^ a topological circuit is defined as a subsection of the chain that, if removed, would leave the topology of the rest of the chain unchanged. In Figure 5E, circuits are easily identifiable as those regions whose arcs do not intersect. In the case of AR, for example, we can observe two neatly identifiable circuits, as was to be expected from our structural subdivision into NR and CR. The situation is different for ER and γ-synuclein (SNCG), where charged contacts tend to bring together the two ends of the chain, making it one undivided circuit. GR, much like AR, tends to create multiple substructures, as highlighted both by inspection of the 3D structure and the by the three circuits visible in the circuit diagram. Given the results of this exploratory analysis of the biophysical nature of short- and longer-lived contacts, it is fair to assume that longer-lived contacts maintain some significance in the formation of transient semistable configuration for IDPs. We will then uncouple the role of such contacts from that of short-lived ones in the context of topological analysis, to increase the signal-to-noise ratio at the level of structural and biological characterization. We will mostly focus on middle and short life contacts, as the sample size of long-lived contacts is often too small for statistical analysis. Moreover, such contacts have a lifetime compatible (or equal) to the duration of the MD runs; we are thus unable to view their topological evolution play out and we cannot assess how dependent their arrangement is from the chosen initial configuration.

**Figure 6:**
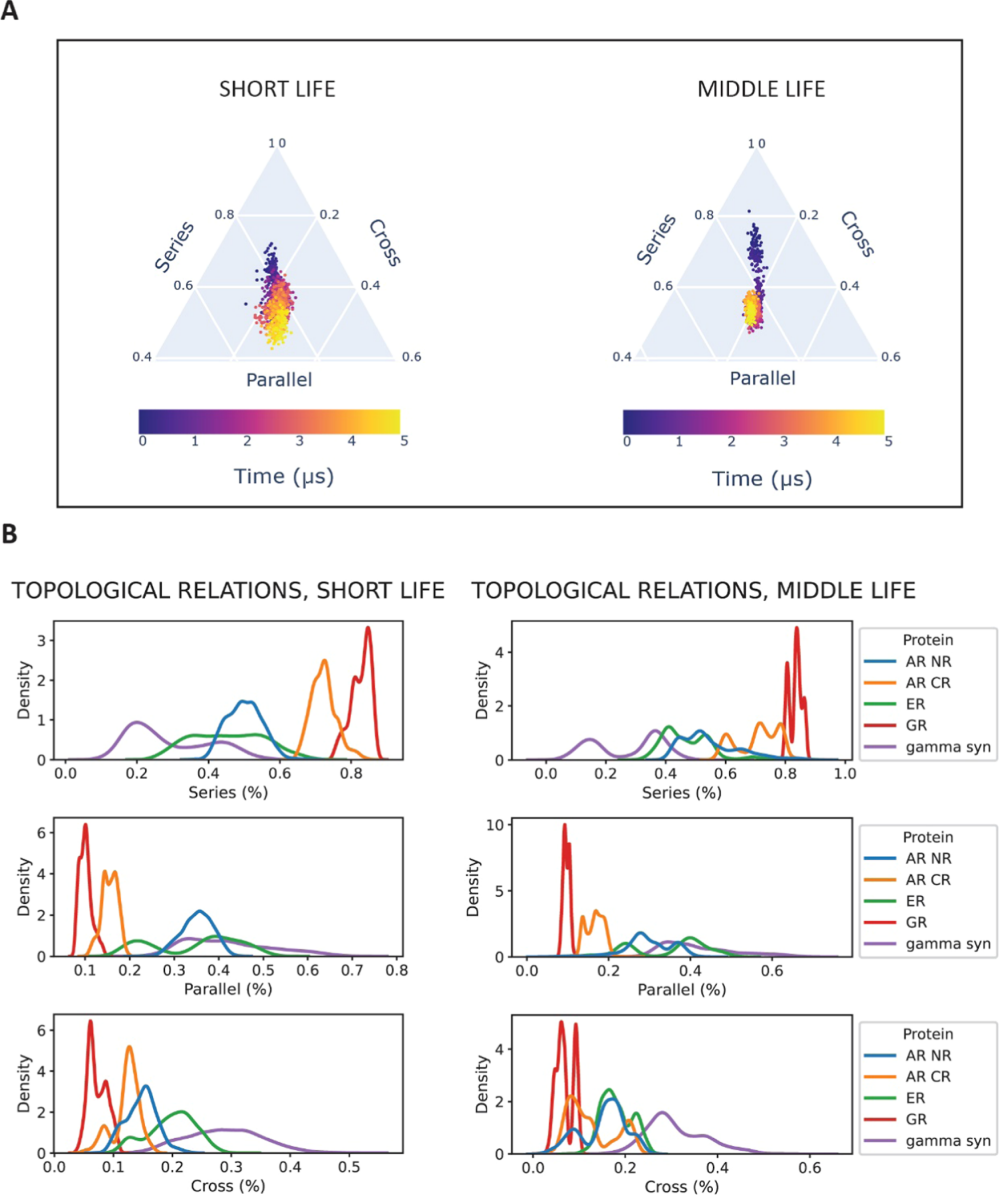
Different timescales of IDR/IDP dynamics can be characterized by different topological makeup. (A) Topological evolution of short and middle life contacts of ER-NTD, MD run 2. The evolution is depicted over the topological landscape, a three-dimensional space where the dimensions correspond to the percentage of series, parallel, and cross contacts. The number of S, P, and X contacts was normalized so that the sum is equal to 1. (B) Distribution of topological relations over the three MD runs for each protein. In most cases, middle life distributions show more peaks, indicating that the system is exploring more transient states.

One of the main advantages of using the CT framework for the representation of such complex configurations is the reduction in dimensionality. As a first-order analysis, we can characterize any configuration by the percentage of S, P, and X relations which contacts at a certain time *t*_*i*_ occupy. This procedure presents us with the nontrivial advantage of being able to represent configurations as coordinates in a 3D space, which from now on we will call the topological space (the triangular plots in Figure 6A). Even with this substantial simplification in terms of configurational complexity, the patterns created during IDP evolution over the topological space are extremely rich in information. One can, first and foremost, identify the number and boundaries of transient states, which appear as globular patterns in the triangular plot. Moreover, one can detect an overall direction in the configurational search, and quantify its topological evolution. The first observation that becomes apparent inspecting such plots is that the trajectories created by middle life contacts generally present a higher number of transient states as opposed to those created by short life contacts, indicating that, indeed, IDPs experience a multimodal topological evolution, which is timescale-dependent. This phenomenon becomes also apparent if we plot the one-dimensional distribution of each topological relation, for each protein under study (Figure 6B). We can observe how middle life distributions have more local maxima, indicating the transient occupation of multiple states. Moreover, even with this first-order analysis, we could already envision two subgroups with different behavior among the NHR-IDRs under study and γ synuclein (SNCG), a synuclein protein used in this study as an example of a non-NHR IDP; AR CR and GR display narrower and more peaked distribution, a sign of a much more stable structure subject to smaller fluctuations. On the other hand, AR NR, ER, and SNCG present spread distributions, often overlapping, indicating a very fast-paced, plastic evolution. We will go in depth exploring these patterns with our suggested higher-order topological analysis.

## Characterization of IDP Conformational Trajectories in the Topological Space

**Figure 7:**
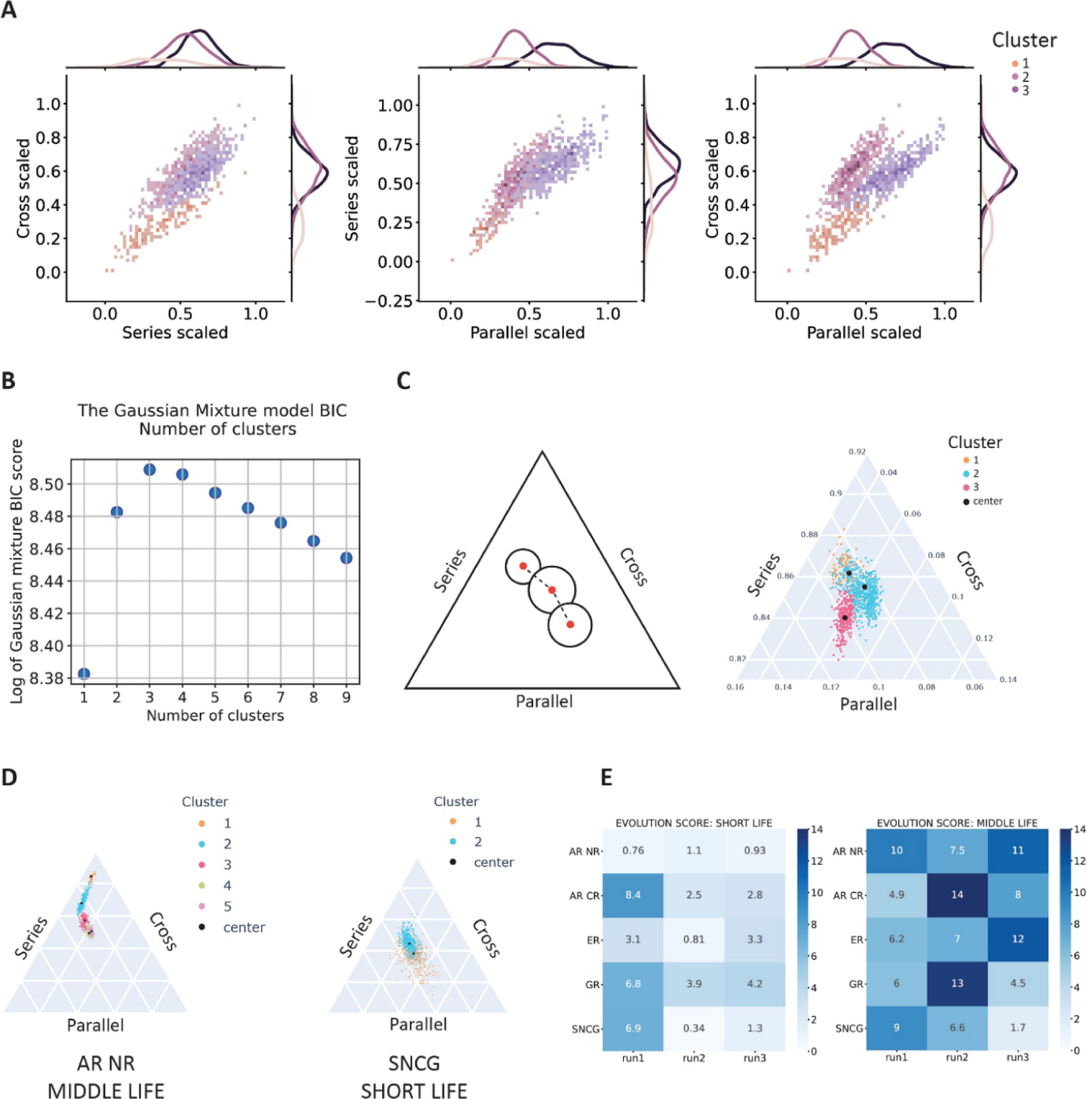
Topological evolution of IDRs/IDPs can be tracked and quantified by identifying intermediate topological states. (A) Scatter plot and one-dimensional distribution of the topological coordinates (in terms of number of series, parallel, and cross contacts) for GR-NTD short life contacts The Gaussian mixture (GM) clustering algorithm identified three clusters, corresponding to three different topological transient states. (B) Maximum BIC score indicates the ideal number of clusters for the dataset, in this case, GR-NTD MD run 2, short life contacts. (C) Left: graphical representation of clusters, cluster centroids, and distance between the cluster centroids. Right: representation of the outcome of the clustering procedure displayed in (A) over the triangular topological space. The number of S, P, and X contacts was normalized so that the sum is equal to 1. (D) Two examples of clustering over the topological space, one corresponding to high evolution score (AR NR, middle life) and one corresponding to low evolution score (SNCG, short life). (E) Evolution score calculated over the whole dataset, subdivided into short and middle life regimes.

The conformational space sampled by IDPs can be seen as a quasi-continuum of rapidly interconverting structures.^40^ The topological evolution of such proteins escapes the traditional method of characterization, which is generally meant for funnel-like folding pathways rather than a flat energy landscape such as those characterizing IDP dynamics.^26^ The dynamic behavior of IDPs is strongly related to their flexibility and versatility,^41^ and therefore the ability to characterize their interconversion between different topological states is key for understanding their function. As a result of our intuitive representation of IDP trajectories over the topological space, we are now in condition to characterize their dynamic hopping between conformations. The first step in this direction is the identification and segmentation of the trajectory into different topological states. In order to do so, we performed clustering over the three-dimensional topology state, where the variables are the number of P, S, and X relations in each configuration. As pointed out by Grazioli et al.,^42^ accurate clustering procedures over the IDP conformational space can prove to be quite challenging because of the vast and flat energy landscape characterized by innumerable microstates corresponding to roughly the same energy.^43^ For this reason, we opted for the more expensive Gaussian mixture clustering algorithm, instead of the more popular and fast option, *K*-means. Modeling the conformational states as a superposition of intersecting 3D Gaussian distributions yields a more natural partition of the topological space (Figure 7A), rather than a distinction based on the 3D distance between coordinates (Figure S5). A rather crucial parameter for our analysis is the number of clusters in which to segment the configuration space. In order to provide an objective metric for it, we relied on the optimization of the Bayesian Information Criterion (BIC) score.^44^ The BIC score is calculated for the data by fitting them for a varying number of clusters (Figure 7B). The number of clusters that provides the highest BIC score is picked for further analysis. We found that feeding the algorithm a different value of parameters such as reg_covar (the non-negative regularization added to the diagonal of covariance) might result in a different number of clusters selected by the BIC score. Here we report results for the default value of reg_covar = 1.0 × 10^–6^. However, results for other values are reported in (Figure S6), together with a summary table of the number of clusters detected for each MD run and each protein (Tables S1–S6). An example of such a clustering procedure is reported in Figure 7A,C, for GR short life contacts, MD run 2. As previously mentioned, clusters (or topological states) appear as globules on the normalized triangular topological space (Figure 7C). By inspecting such patterns, it becomes apparent that some trajectories happen to be more elongated, covering a higher portion of the topological space, and show a higher tendency to hop between states than others (Figure 7D). In order to quantify this tendency, and also to provide a metric to characterize the quasi-continuum interconversion between states typical of IDP dynamics, we defined a new parameter. Given two clusters, C_1_ and C_2_, the evolution score *E*_21_ is given by , where *s*_1_ and *s*_2_ are the spread of clusters C_1_ and C_2_, respectively, and *d*_21_ is the 3D distance between the centroid of C_1_ and C_2_. Since by choice of algorithm our clusters are described by Gaussian distributions, the centroid corresponds to the mean of the Gaussian. This definition is generalized for the case in which we have more than two clusters by summing each contribution *E*_*ij*_ to the total evolution score *E*, where C*_i_* is the cluster subsequent to C*_j_* from the point of view of temporal evolution. Other empirical definitions of the evolution score were also tested; the results can be found in Figure S7. Although our general conclusions do not change, we found that the formulation described above provided the best match to the visual behavior of the trajectories in the topological space. What does this metric portray, intuitively? We can expect a trajectory characterized by a low *E* value to be very globular in nature, with few, wide clusters, that tend to occupy the same portion of the topological space. On the other hand, high *E* values are yielded by trajectories that are very narrow and directional, characterized by a substantial exploration of the topology space, often with multiple clusters occupied in a row (Figure 7D). The results of such analysis are of course very much dependent on which part of the conformational ensemble is the IDR/IDP exploring with one particular trajectory, and therefore several such trajectories should be explored in order to make IDP-specific statements. However, even with our limited sampling, we can already deduce some general observations, from the results in Figure 7E. First of all, we see that, in most cases, scores for short life topology are lower than those for middle life topology. This finding quantifies our previous intuition, which was, longer-lived contacts tend to occupy a higher number of topological states, and cover larger portions of the topological landscape. This conclusion could help identify key contacts for semistable IDP structures, as well as functional folding-upon-binding configurations.^45^ This trend is particularly accentuated in AR NR, SNCG, and ER, which show a consistent increase of *E* score from short to middle life, for all runs. These three IDR/IDPs are also the ones showing the overall minimum scores for short life. This result suggests very wide clusters, characterized by a very unstable, plastic structure. The bigger the spread, the less defined the underlying structure. Moreover, the effect we observe might be dependent on size, as these three specimens are the smallest in our dataset; the shorter the chain, the easier it might be to explore the configurational space with very wide clusters. However, once the short-lived contacts are filtered out, a directional topological evolution appears, which is not very dissimilar from that of larger proteins.

On the other hand, GR and AR CR seem to maintain more or less the same range of *E* scores for both short and middle life, indicating a certain topological symmetry for what concerns different temporal modes of evolution, and most likely persisting semistable topological structures. In the case of these two IDRs, we even observe sometimes a decrease in evolution score going from short to middle life regime (run 1). These simple considerations already allow us to cluster together proteins displaying similar patterns of dynamic behavior in short and middle life modes. Such an approach, coupled perhaps with relaxation times experimentally derived from NMR, or some other techniques for enhanced sampling, could provide invaluable for the quantification of IDP configurational dynamics.

## Topological Strings and Sequence Alignment

**Figure 8:**
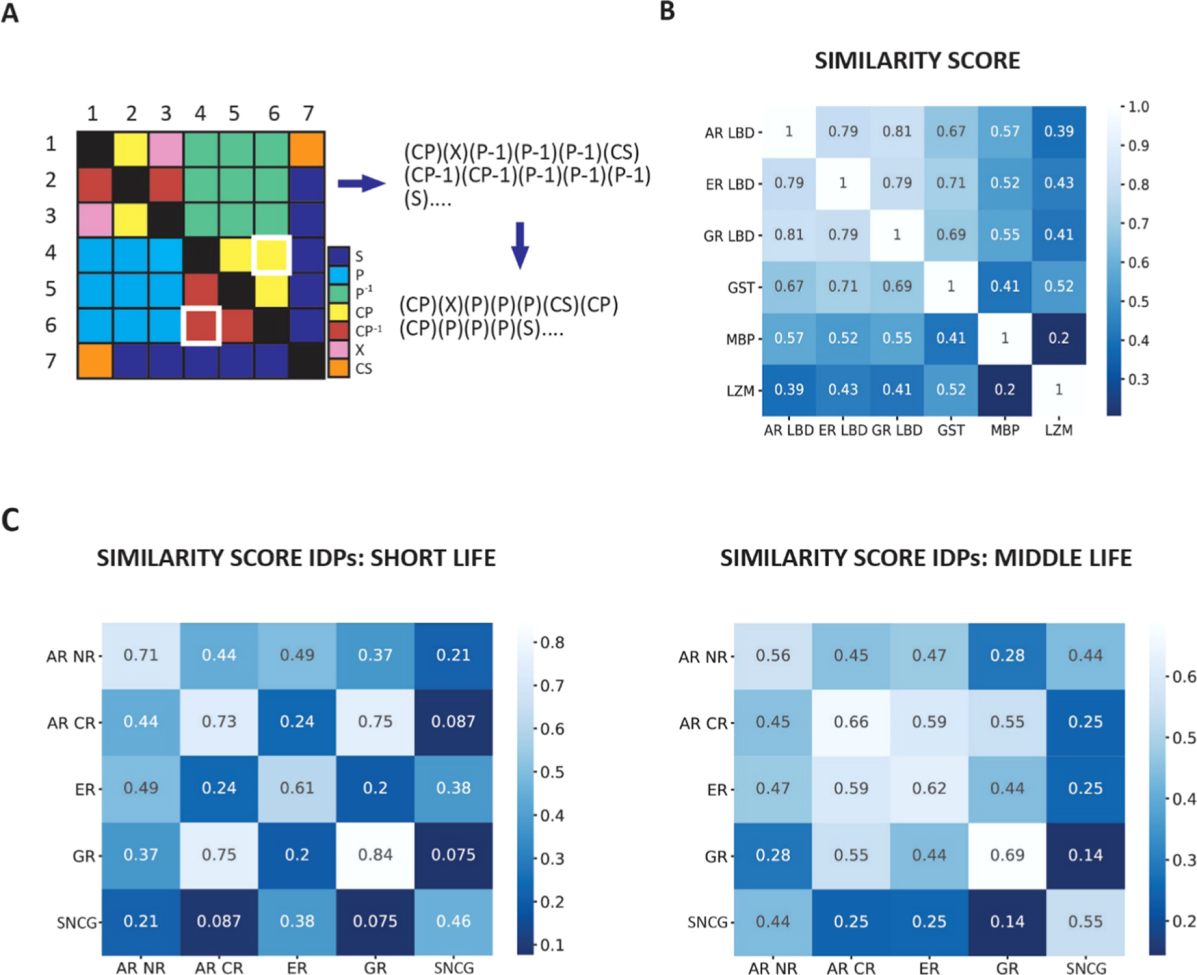
Circuit topology, expressed in form of topology strings, can be used to measure the similarity between different IDPs/IDRs. (A) Schematics representing the linearization procedure necessary to go from topology matrix to topology string. In the string, topological relations such as P^–1^ and CP^–1^ are incorporated with P and CP since they all represent the same topological arrangement. The topology matrix is symmetric. The elements highlighted in white, (6,4) and (4,6) indicate the same topological relation between contact pair 4 and 6. It is easy to see how linearizing the matrix row by row accounts for all four nearest neighbors of each matrix element, along both rows and columns. Take element (4,6): its nearest neighbors on the string will be (4,5), CP and (4,7), S. However, when we get to its specular representation on the other side of the diagonal (6,4), we see that its nearest neighbors in the string will be P and CP^–1^, which were the nearest neighbors of element (4,6) along its column. We are therefore accounting for the proximity of elements on both rows and columns. (B) Pairwise similarity scores for model proteins and AR, GR, and ER LBD. The scores were obtained by alignment of strings representing protein native topology. (C) Pairwise similarity scores for IDRs/IDPs. The scores were obtained by aligning strings corresponding to the topology reached by the protein in the centroid of the last occupied topological state during the MD run. Scores obtained for all three MD runs are averaged into one value. On the diagonal, we have the average similarity score obtained by comparing the three runs of one protein.

Functionally similar IDPs often have no significant sequence similarity.^46^ Moreover, the lack of stable tertiary structure complicates the picture further, making it challenging to compare such proteins by structure alignment techniques. The issue of functional classification of IDPs was recently tackled by a technique called sequence charge decoration (SCD),^33^ which relies on the charge patterning of the sequence, which serves as an indication of the ensemble average distance between pairs of residues.^47^ Here, we propose a method to identify similar topological blueprints between different IDPs, which can be applied to any transient conformation, without relying on averaging conformations over the ensemble. We create topological strings out of specific IDP conformations which are suitable for sequence alignment, overcoming the issue of little to no sequence similarity. In order to explain this topological alignment procedure, we have to introduce the concept of topology matrix (Figure 8A). So far, we have only considered the overall number (or percentage) of S, P, and X relations characterizing a certain conformation occupied by the protein at time *t*. However, we can also consider the patterning with which these relations appear in the chain. To do this, we consider an *N* × *N* matrix, where *N* is the total number of contacts in the chain at a given timepoint. Each contact (formed by residue *i*, *j*) is numbered based on the indexing of its first contact site (residue *i*). Each element in the matrix is filled in based on the topological relation between the relevant pair of contacts. We can see two versions of the P and CP relation in the matrix, that is, inverse P and CP (P^–1^, CP^–1^); this specification is made because parallel is not a symmetric relation: when contact A is enveloped by contact B, we say that A is in parallel relation with B. However, B is now enveloping contact A, not being enveloped by it. We say therefore that B is in inverse parallel relation with A. However, these two wordings refer to the same topological arrangement between two contacts, so for the sake of topological sequence alignment, only the labels P and CP will be used for all cases. To retrieve sequences out of topology matrices we perform a simple matrix linearization. Linearizing by rows or columns makes no difference in this case since the matrix is symmetric. Thanks to symmetry, linearizing by rows means to account for the locality of matrix elements along both rows and columns. This fact can be clarified by looking at the elements highlighted in white in Figure 8A. Take element (4,6): linearizing by rows, its nearest neighbors are CP on one side and S on the other. Its nearest neighbors along columns, P and CP, are at this stage not accounted for. However, when we get to the symmetric representation of the same element, (6,4), we see that its nearest neighbors along rows are now P and CP. In this way, the locality of topological relations is accounted for along both rows and columns, regardless of our choice of linearization along rows or columns. In this way, we obtain topology strings from any conformation. We can couple this technique with the clustering procedure presented previously in this study, in order to pick meaningful configurations for our analysis. For our exploratory comparative analysis, we picked the centroid of the last cluster occupied by each protein in each 5 μs MD run. In this way, we could calculate a similarity score for the pairwise alignment of three sequences for each IDR/IDP, resulting in a 15 × 15 similarity matrix (Figure S8). The choice in terms of cluster is by no means unique, and the analysis could be generalized to any state occupied by the IDP trajectory. We calculated the similarity score in two different ways: by global sequence alignment, as provided by the Biopython Pairwise2 module (Figure 8B,C), and by the difflib SequenceMatcher class in python (Figure S9). Both methods yield the same patterns of similarity. In order to test the capability of the method to retrieve structural similarity between related proteins, we tested it over six non-IDPs, three evolutionary related regions (the LBD of AR, ER, and GR) and three unrelated model proteins (maltose binding protein, glutathione *S*-transferase, and lysozyme). The results can be found in Figure 8B, where the highest similarity scores are indeed found among LBD of hormone receptors. Subsequently, we applied this analysis to the IDRs and SNCG, for short and middle life trajectories. Figure 8C presents an average similarity score over the three runs for each protein. We see that, despite the natural heterogeneity of such system, we see a picture emerge that is compatible with the results obtained so far by looking at the dynamical properties of the topological evolution. GR NTD remains the most stable IDR in our dataset, scoring the highest similarity scores within its three runs. Also, GR and AR CR score relatively high in similarity for both short and middle life. ER is most similar to AR NR and SNCG for short life topology. However, for middle life, ER scores relatively high, behaving similarly to AR CR and GR. This dual behavior is in perfect agreement with the results obtained by conformational diffusion analysis. SNCG records the lowest scores overall in the matrix, which is unsurprising since it is functionally very different from the NHRs. However, it does score relatively high with AR NR, also for what concerns middle life, indicating that these two systems share similarities in their topological behavior.

## Discussion

The elusive structural nature of IDPs makes them a very challenging target for homology and functional classification. However, there is growing evidence that common functions of disordered regions and proteins can be found even across evolutionary distant organisms.^48,49^ The recent development of computational and theoretical tools has significantly enhanced our understanding of disorder in proteins.^15^ Molecular dynamics simulations, often coupled with experimental assays, provided new insight into IDP conformational search and ensemble.^31,32,50^ Topology-based modeling^45^ and machine learning techniques^42,51,52^ proved to be invaluable in the characterization of IDP configurational space, often due to their ability to reduce the dimensionality of the system to a few meaningful coordinates and metrics. However powerful, machine learning models are still very dependent on the quality of data and data representation they are fed. The features extracted by circuit topology have the potential to offer such data representation. We reduced the problem to its topological coordinates, offering various types of analysis, ranging from the characterization of the conformational evolution in the topological plane to the topological content itself, which can be quantitatively characterized and used for comparison.

Concerning our dataset, we can summarize a few interesting findings. Traditional methods such as disorder prediction with PONDR and solvent accessibility analysis suggested a lower level of disorder for AR CR and GR with respect to the rest of the dataset. This finding was corroborated and expanded by circuit topology analysis, which found consistent similarities in dynamic behavior and topology for these two IDRs. Multi-timescale analysis revealed that these two IDRs tend to maintain the highest topological coherence between short and middle life modes. AR CR and GR also score the highest in terms of self-similarity among runs (Figure 8C). All of these data depict a picture of a higher relative structural stability for these regions, which are also quite different from the rest in terms of topological makeup. From Figure 6B, we see that AR CR and GR score consistently higher in series relations and lower than the rest in parallel and cross. This finding is not surprising when taken in context with the rest of the analysis; generally speaking, IDPs have higher cross and parallel relations with respect to proteins with a stable tertiary structure. This difference is due to the principles of protein folding and assembly: folded proteins tend to favor local connections first, and form subdomains containing these local elementary structures.^53^ Contacts within a domain will then be in series with contacts within a different domain, or region, because they are shielded and there is no interaction. In IDPs, on the other hand, this happens to a lesser extent, as stable structures are seldom created and interaction remains very dynamic at all times. Therefore, the high percentage of series in AR CR and GR might indeed indicate the formation of semistable structures. This conclusion is also supported by the circuit diagram in Figure 5E: AR CR and GR do not present, like the other IDP/IDRs in the study, the tendency to have charged contacts bringing together the ends of the chain, but rather a more structured circuit structure, potentially indicating the formation of highly connected subdomains.

Multiple sequence alignment found insignificant similarities in the NHR NTD presented in this study, as is often the case with IDRs/IDPs. However, relatively higher matches between ER, AR CR, and GR. Circuit topology analysis depicted a much more nuanced picture for ER, which displays a high heterogeneity and asymmetric behavior with respect to short and middle lifetime scales. While we do find significant similarity in topology sequence matching with AR CR and GR for middle life, in short life ER reveals a very dynamic behavior which makes it easier to cluster it together with AR NR and SNCG. Finally, AR NR and SNCG display very similar behavior across the board, in spite of being evolutionarily unrelated. They show very plastic evolution, with less tendency to form semistable structure, and with significant asymmetry when it comes to topological evolution in short and middle life modes. It has been hypothesized that some IDPs present residual structures which modulate the entropic cost of folding facilitating binding thermodynamics.^45,54^ However, other cases suggest that increased local structure in the unbound state of IDPs might actually reduce binding rate,^55^ stressing the importance of disorder for functionality and versatility of these proteins. It is possible that we are now observing these two opposite tendencies in our dataset, with AR CR and GR presenting residual structure, AR NR and SNCG having a higher level of disorder and plasticity, and ER being somewhere in the middle.

The analysis presented in this paper explores the possibility of comparative IDP analysis by use of the circuit topology framework coupled with molecular dynamics simulation. While challenges related to the vast and flat energy landscape and conformational space of IDPs remain, we believe CT could be an invaluable framework for data processing and visualization to tackle these systems. Moreover, several elements of the presented pipeline can easily be coupled to other, well-established topological frameworks, in order to enhance their predictive capabilities and provide a more complete description of protein structure. We exemplify here this concept by discussing possible applications of the dynamic CT pipeline to a successful mathematical tool for topological analysis of biomolecules, persistent homology.^56,57^

Persistent homology is a branch of algebraic topology that has allowed in recent years to define topological fingerprints (MTF) of proteins,^57,58^ and reached high-performance predictions in a variety of tasks, protein classification,^58^ protein/ligand binding affinities,^59−61^ protein/protein interaction energy,^62^ protein folding and stability changes upon mutation,^63^ and drug virtual screening.^60,64^ To summarize, persistent homology concerns itself with the identification of topological properties of a given space, such as holes and voids, and to quantify how long these features persist over different spatial scales. This process, known as filtration, allows researchers to examine the structure of a space at various resolutions and understand how it changes as features appear and disappear. At a given resolution, these topological properties are expressed in terms of Betti numbers, indicating the number of connected components, tunnels, cavities, etc.^57^ The CT formalism was previously applied in the context of extended persistent homology:^65^ specifically, CT relations were used for the characterization of simplicial complexes, which constitute the mathematical construct used to represent the topology of a space for PH characterization. It is noteworthy to mention that spaces characterized by the same Betti numbers might correspond to different configurations in the CT space, as CT relations are mostly concerned by the reciprocal arrangement of connected components of a space rather than the number of connected components specifically. Therefore, CT relations might be used to discern between different configurations in the formalism of PH, if the problem at end requires for it. Moreover, various methods described in this paper could be coupled to PH in a variety of ways. For example, Betti numbers could be used to select which configurations to plot in the 3D topological space created by CT parameters (Figure 6A). One could decide to plot only those configurations that are topologically equivalent (identified by the same Betti number) and follow their evolution in the CT space. Alternatively, one could choose to plot only those configurations whose topological features display a certain persistence or to observe only configurations at a given resolution, provided by the filtration parameter. Moreover, multiscale persistent functions such as, for example, multiscale persistent entropy (MPE),^66^ can be used to assign specific indexes to any given configuration, such as a protein structure index (PSI). Such an index could be easily plotted as a color map on the triangular CT space, to observe how configurations evolve in terms of disorder.

Various additions have been made on the persistent homology framework to ensure retention of fundamental biological, chemical, and geometric characteristics. Examples of these are multiscale and element-specific persistent homology (ESPH),^63^ weighted and localized weighted persistent homology (LWPH).^67^ These methods could be used for the selection of biologically meaningful contacts to plot with our circuit analysis (Figure 5E) while leveraging on this type of visualization to identify the underlying reciprocal structure of these contacts.

Topological features extracted by persistent homology have seen very successful machine learning applications,^59−63^ displaying the potential of topology for predictive analysis. Similarly, CT could easily be coupled with enhanced sampling, clustering, and various machine learning and network analytics methods, to provide a new topological perspective on intrinsic disorder.

## Methods

### Three-Dimensional Structure Prediction of NHR NTDs

There are no resolved structures of the N-terminal transactivation domains of the nuclear hormone receptors deposited on the Protein Databank (PDB) due to their disordered nature. To initiate our studies from computationally efficient initial structures, the three-dimensional structure of the NTDs was modeled using the I-TASSER server,^29^ the best protein structure prediction method according to the Critical Assessment of Protein Structure Prediction (CASP) community.^68^ I-TASSER employs a hierarchical approach to protein structure prediction and structure-based function annotation. This approach is either comparable to or outperforms AlphaFold^69^ and RoseTTAFold^70^ in predicting the experimentally measured secondary structure content of disordered proteins included in this study, based on the available data.^28^ To further optimize the initial structures, energy minimization steps using the steepest descent method were performed followed by conjugate gradients with an ff99SB all-atom force field to perform a total of 100,000 steps per protein construct using GROMACS software packages.^71^

### Molecular Dynamics

For this study, 5 μs molecular dynamics (MD) simulations were performed on the energy-minimized structures acquired by the structure prediction pipeline in the previous section. To reduce computational costs, the SIRAH coarse-grained force field^72^ for proteins was used in combination with a WT4 explicit coarse-grained water model. The proteins were mapped to a coarse-grained representation according to the standard SIRAH mapping. A rhombic dodecahedron box was used to dissolve the structure by adding WT4 water molecules. Electroneutrality and physiological concentration of salt were achieved by replacing the corresponding amount of water molecules with NaW and ClW (coarse-grained representations of Na^+^ and Cl^–^ ions, respectively). All coarse-grained systems were minimized using the steepest descent algorithm before a 5 ns NVT equilibration, 5 ns NPT equilibration, and an NPT production run. The leapfrog integrator with a 20 fs time step was used throughout. Protein beads were constrained with the LINCS algorithm^73^ during the equilibration, and no constraints were employed during the minimization and production steps. The temperature was kept at 310 K with a velocity rescale thermostat,^74^ and the pressure at 1 bar with the Parrinello–Rahman barostat. τ_T_ for the thermostat was set to 1.0 ps during the equilibration phases and to 2.0 ps during the production. τ_P_ for the barostat was set to 10.0 ps during both the NPT equilibration and the production. Both nonbonded cutoffs (van der Waals and short-range electrostatics) were set to 1.2 nm. Long-range electrostatics were treated with the particle mesh Ewald (PME) method with a 0.2 nm grid spacing during the equilibration and 0.25 nm during the production. Nonbonded interactions were calculated using a 1.2 nm cutoff neighbor list, updated every 25 steps (in the production and the NPT equilibration) or 10 steps (in the NVT equilibration). Both energy and pressure dispersion corrections were applied. Periodic boundary conditions and the minimum image convention were used. Snapshots were collected every 1000 steps (20 ps). All simulations and subsequent analyses were carried out with GROMACS 2020.^71^

### Order–Disorder Prediction

Structural disorder was analyzed using the PONDR^27^ webserver, and raw data obtained from the server and plots were made using OriginPro 2021 (OriginLab Corporation, Northampton, MA).

### Preparing the Structures for Circuit Topology Analysis

After the trajectories of the systems were retrieved, atomic positions of amino acids were generated from the location of CG beads. Backmapping was done using the sirah_vmdtk.tcl plugin, followed by 100 steps of steepest descent and 50 steps of conjugated gradient minimization in vacuum using the sander module of AmberTools.^75^ This procedure was robust and independent of the fine details of the backmapping library. The obtained atomistic coordinates were used for circuit topology analysis.

### Timescale Analysis

Contact maps were exported from our custom-made Circuit Topology Python 3 tool.^76^ In our CT tool, contacts are identified by means of two cutoffs, one relative to the spatial distance *r* between atoms (4.5 Å), and one relative to the number of atom pairs that need to be found at a distance less than *r* to consider the two residues in contact. Contact maps were then processed to extract the contact lifetime distribution of a specific MD run. Each contact is identified by the unique pair of residues forming it; the same contact can form and break multiple times in an MD run, therefore its lifetime is not unique. We picked the maximum lifetime for each possible pair of residues to build the contact lifetime distribution, under the assumption that a contact will contribute the most to the structure when it persists the longest in the run. The lifetime data were fitted by NaiveKDE from the KDEpy library,^77^ a naive computation of a kernel density estimate, in order to extract the underlying distribution. The log–log plot of such distributions can be seen in Figures 4C and S3. The log–log distribution was then fit to a power law
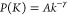
with the least square fitting procedure (Scipy.stats.linregress^78^). Fitting was performed over subsequently larger subsets of data, starting from the first three data points and incrementing the set one datapoint at a time. The quality of each fit was then evaluated by calculating the coefficient of determination *R*^2^. This stepwise fitting and evaluation procedure was done in order to set the boundaries for the applicability of the power law and identify thus two different timescales for IDP dynamics (short and middle life regime). We set two thresholds for *R*^2^ values (Figure 4D): we set the end of the short life regime when *R*^2^ displays and initial drop below *t*_1_ = 0.8. The second boundary is retrieved from the datapoint where the *R*^2^ curve rises above *t*_2_ = 0.3, after reaching its global minimum. These two thresholds were set empirically based on the good visual agreements between different IDPs (Figure S4). Contacts were then assigned to either short, middle, and long life regimes based on their lifetime and the temporal threshold found via *R*^2^ evaluation. Two contact maps were created, one for short and one for middle life contacts, while long life contacts were discarded.

### Circuit Topology Analysis

Contact maps calculated for specific time regimes were loaded as filters in our CT tool,^76^ in order to calculate topological parameters selectively for the chosen dynamic mode. Topological relations are calculated from residue–residue contacts, by assigning an index to contacts based on the order with which the first residue in the contact residue pair appears along the chain, scanning it from left to right.

CT relations were assigned based on the mathematical relations summarized below
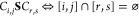

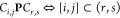

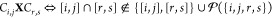

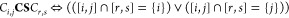

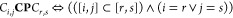
 denotes the powerset, i.e., all subsets of a set including the null set (⌀). *C*_*i*,*j*_ and *C*_*r*,*s*_ indicate contacts formed, respectively, by the *i*th and *j*th, and by the *r*th and *s*th contact sites. Contact indexes (*i*, *j*, *r*, *s*) were assigned by scanning the chain from the left end to the right end. For more information about the formalism, we invite the reader to refer to Mashaghi et al.^20,21^ and Schullian et al.^79^ Topology matrices store then the topological relation between each pair of contacts. Both CT relations and topology matrices were exported for further analysis.

### Clustering

Clustering was performed by means of scikit-learn,^80^ a library for machine learning in Python. CT relations were preprocessed for clustering using MinMaxScaler (scaling values from 0 to 1). Clustering was performed by following a Gaussian mixture model probability distribution (mixture.GaussianMixture), by inputting a number of clusters ranging from 0 to 10. Results reported in the paper were calculated with the following input parameters: number of initializations to perform: 100, convergence threshold: 1 × 10^–4^, maximum number of iterations to perform: 10 000, non-negative regularization added to the diagonal of covariance: 1 × 10^–6^. Results for different regularizations can be found in Figure S6. The ideal number of clusters for each dataset was then picked by optimizing the Bayesian Information Criterion (BIC) score.^44^ Centroids of the clusters were calculated as 3D mean the cluster data points. The spread of the cluster was evaluated by calculating the mean of the Euclidian distance between the data points in the cluster and the centroid.

### Sequence Alignment and Similarity Score

The similarity score between topology strings was calculated with two different procedures, to test the robustness of the method and finding the least expensive computational method:

- Global alignment with Bio.pairwise2 (Biopython^81^): the module provides pairwise sequence alignment using a dynamic programming algorithm. Global alignment finds the best concordance between all characters in two sequences; the score thus found was then normalized by multiplying it by 2/(*l*_1_ + *l*_2_), where *l*_1_ and *l*_2_ are the lengths of the first and second sequences, respectively. Such an alignment procedure is symmetric, which means that the similarity score does not depend on the order in which the sequences are fed into the algorithm. Although its many advantages, this method is computationally expensive in terms of memory usage and time. Since we often incurred in memory errors while handling alignment for the largest topology strings, we decided to apply a coarse graining procedure over all topology strings. A comparison between similarity scores with and without coarse graining is presented in Figure S10, for middle life contacts: differences in scores are negligible and do not affect the general conclusions in the study. Coarse graining was performed by assigning a number to each topological relation: S = 0, CS = 1, P = 2, CP = 3, and X = 4. Numbers were assigned following the rationale according to which entangled, interacting topologies like X might weight more than noninteracting one, such as S. Then, we performed a mean over each five subsequent elements of the string, yielding the corresponding element of the new coarse-grained string. Each element was then rounded in order to yield an integer. Sequence alignment was then performed on the coarse-grained string.
- Similarity score calculation with the Python module difflib, SequenceMatcher: this algorithm does not yield minimum edit distance between sequences, but rather finds the longest contiguous matching subsequence, and then recursively applies the same procedure to the rest of the sequence, to the right and to the left of the matching part. This procedure is less precise than global alignment. However, it is faster and does not require any type of coarse graining. The two procedures yield the same general results; the score similarity score in this case is calculated as “quick_ratio” or “real_quick_ratio.”

## Data Availability

The python codes and force field parameters are deposited at: https://github.com/circuittopology/dynamic_circuit_topology.

## Supporting Information Available

Radius of gyration analysis of AR NR, AR CR, and full-length ER NTD (Figure S1); order and disorder-promoting residues content of the chain calculated separately for AR NR and AR CR regions (Figure S2); contact lifetime distribution for IDP/IDRs (Figure S3); coefficient of determination *R*^2^, used to evaluate the goodness of the power law fit performed over subsequent chunks of the contact lifetime distribution (Figure S4); comparison *K*-means and Gaussian mixture (GM) clustering (Figure S5); evolution score results for different clustering parameters (Figure S6); evolution score results for different empirical definitions of evolution score (Figure S7); similarity score for each pair of IDP/IDR in the dataset (Figure S8); similarity score as calculated by SequenceMatcher method (Figure S9); and similarity score (for middle life contacts) calculated with and without coarse graining of the topology strings (Figure S10) (PDF)
