# Supplementary material for: Circuit Topology Approach for the Comparative Analysis of Intrinsically Disordered Proteins
Source: J Chem Inf Model. 2023 Apr 7;63(8):2586–602. doi: 10.1021/acs.jcim.3c00391 (PMC10131221; doi:10.1021/acs.jcim.3c00391)
Supplement: Supplementary file 1 — ci3c00391_si_001.pdf [file ci3c00391_si_001.pdf]

Supplementary Information

Circuit Topology approach for the comparative analysis of intrinsically disordered proteins

Barbara Scalvini[1,2], Vahid Sheikhhassani[1,2], Nadine van de Brug[1,2], Laurens W.H.J. Heling[1,2], Jeremy D. Schmit[3], Alireza Mashaghi[1,2†*]

[1] Medical Systems Biophysics and Bioengineering, Leiden Academic Centre for Drug Research, Faculty of Science, Leiden University, Einsteinweg 55, 2333 CC Leiden, The Netherlands

[2] Centre for Interdisciplinary Genome Research, Faculty of Science, Leiden University, Einsteinweg 55, 2333CC Leiden, the Netherlands

[3] Department of Physics, Kansas State University, Manhattan, KS, 66506, USA

*Email: a.mashaghi.tabari@lacdr.leidenuniv.nl

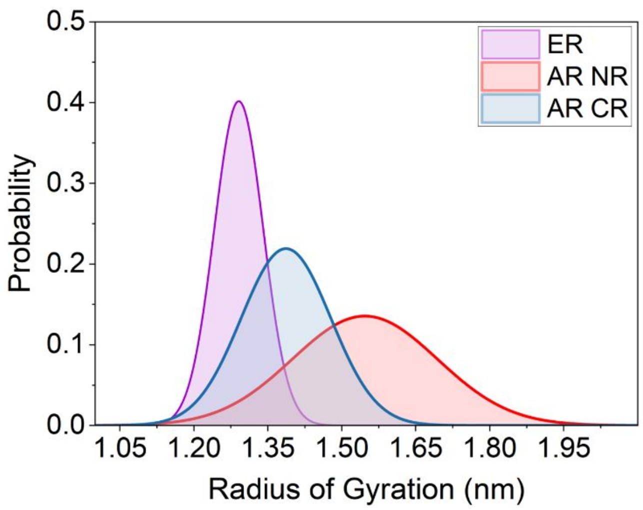

Figure S1. Radius of gyration analysis of AR NR, AR CR and full length ER NTD. Comparing the radii of gyration of CR and NR regions in AR, clearly showed that the CR region is significantly more compact than NR region and both are less compact in comparison to the full-length ER-NTD. The data is normalized by the size of the corresponding chain.

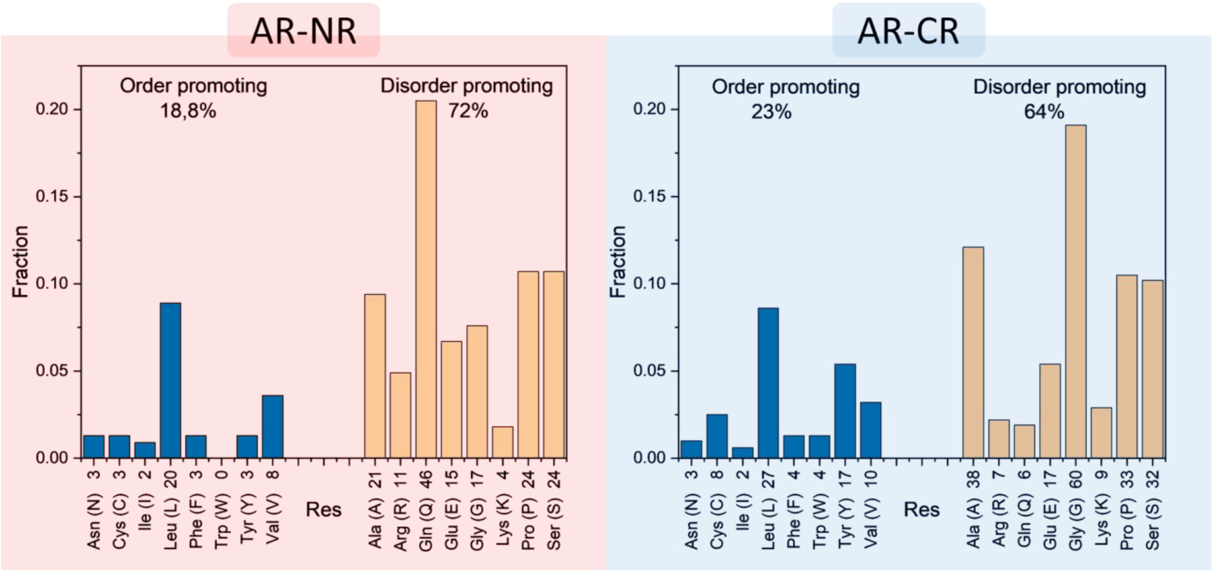

Figure S2. Order (OPR) and disorder-promoting residues (DPR) content of the chain calculated separately for AR NR and AR CR regions. The calculation clearly showed that the OPR content of the NR region was significantly less than the CR.

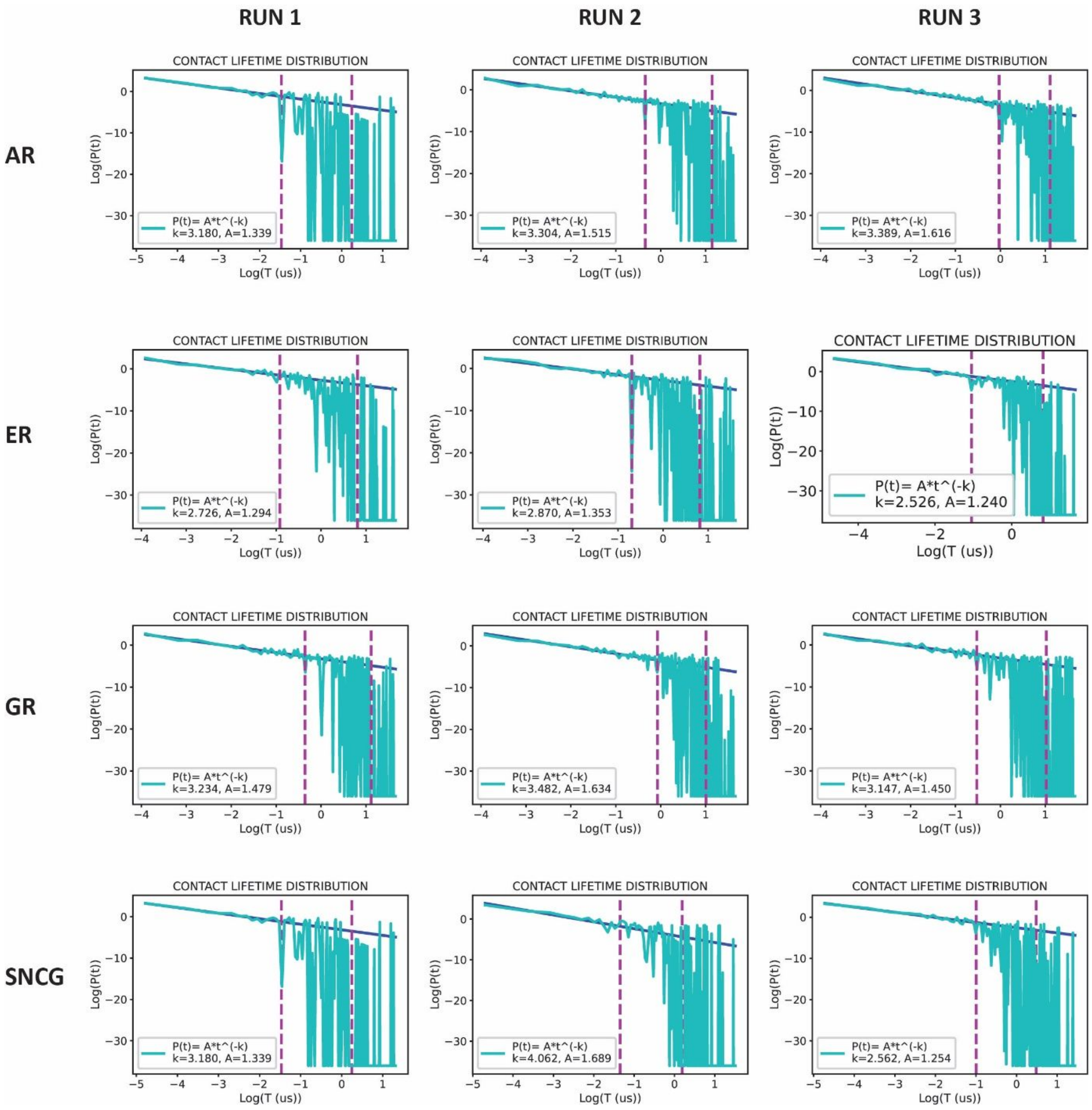

Figure S3. Contact lifetime distribution for IDP/IDRs. Contact life-time distribution, and Power Law fit for all IDP/IDRs, all MD runs. The fit was performed exclusively over short-life contacts, and then extrapolated over the whole range, for visualization purposes.

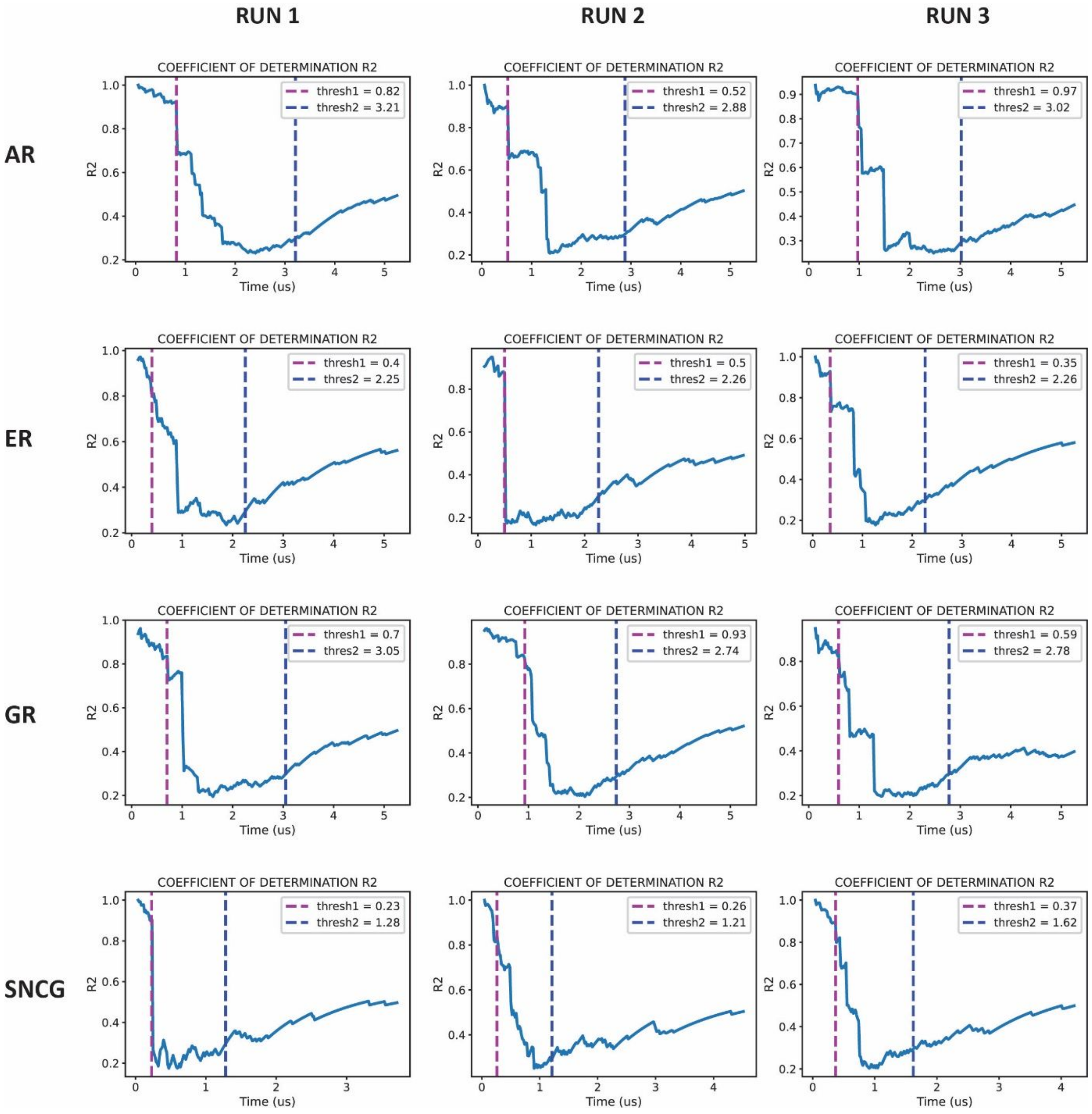

Figure S4. Coefficient of determination R^2, used to evaluate the goodness of the Power Law fit performed over subsequent chunks of the contact life time distribution. The interval was increased by 1 datapoint for each fit iteration.

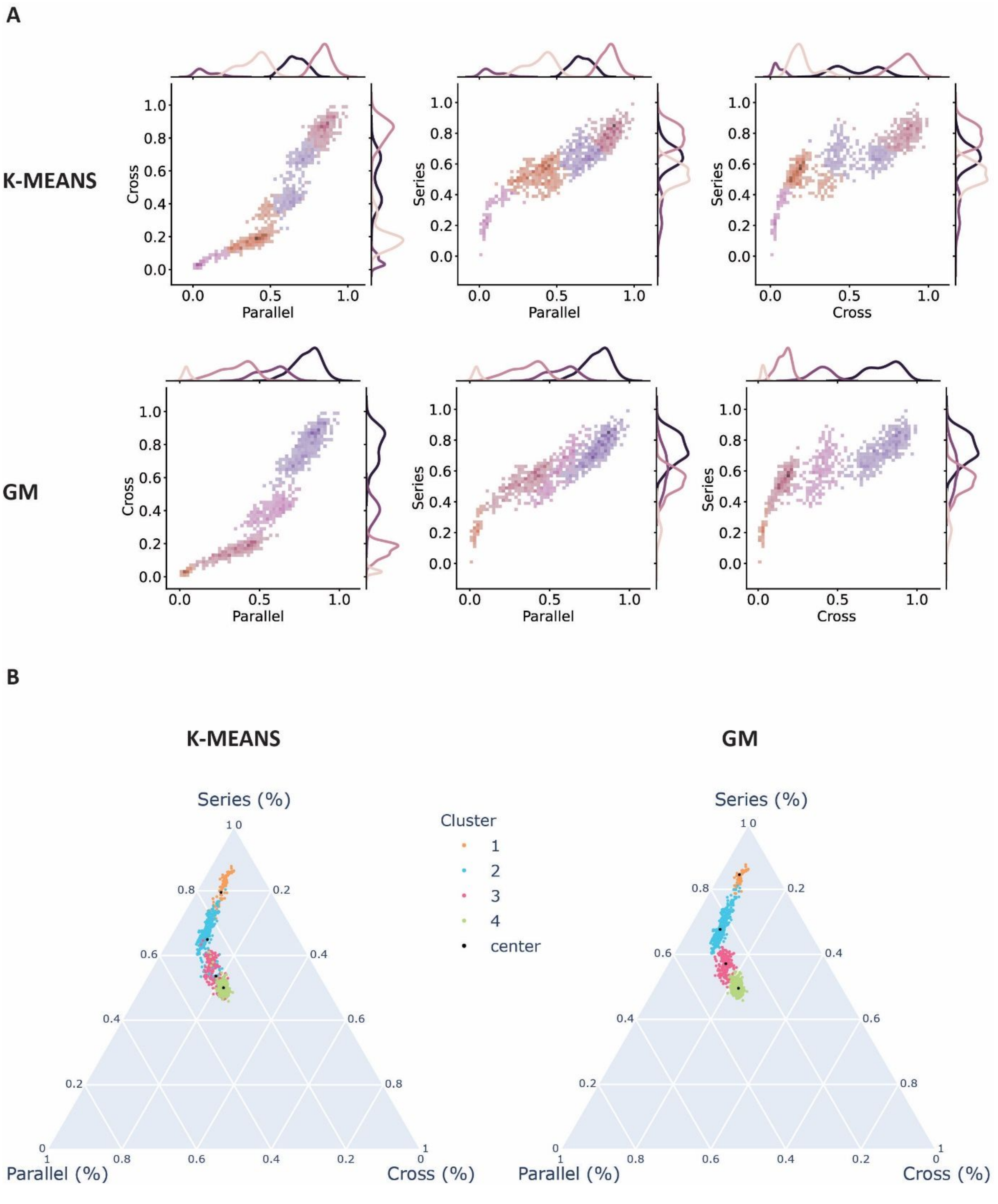

Figure S5. Comparison K-means and Gaussian Mixture (GM) clustering. A Scatter plot and one-dimensional distribution of the topological coordinates (in terms of number of series, parallel and cross contacts) for AR NR, middle life contacts. The clustering was performed by K-means and Gaussian Mixture clustering techniques, for the purpose of comparing their performance. **B** Representation of the outcome of the clustering procedure displayed in A over the triangular topological space, for K-means and GM methods.

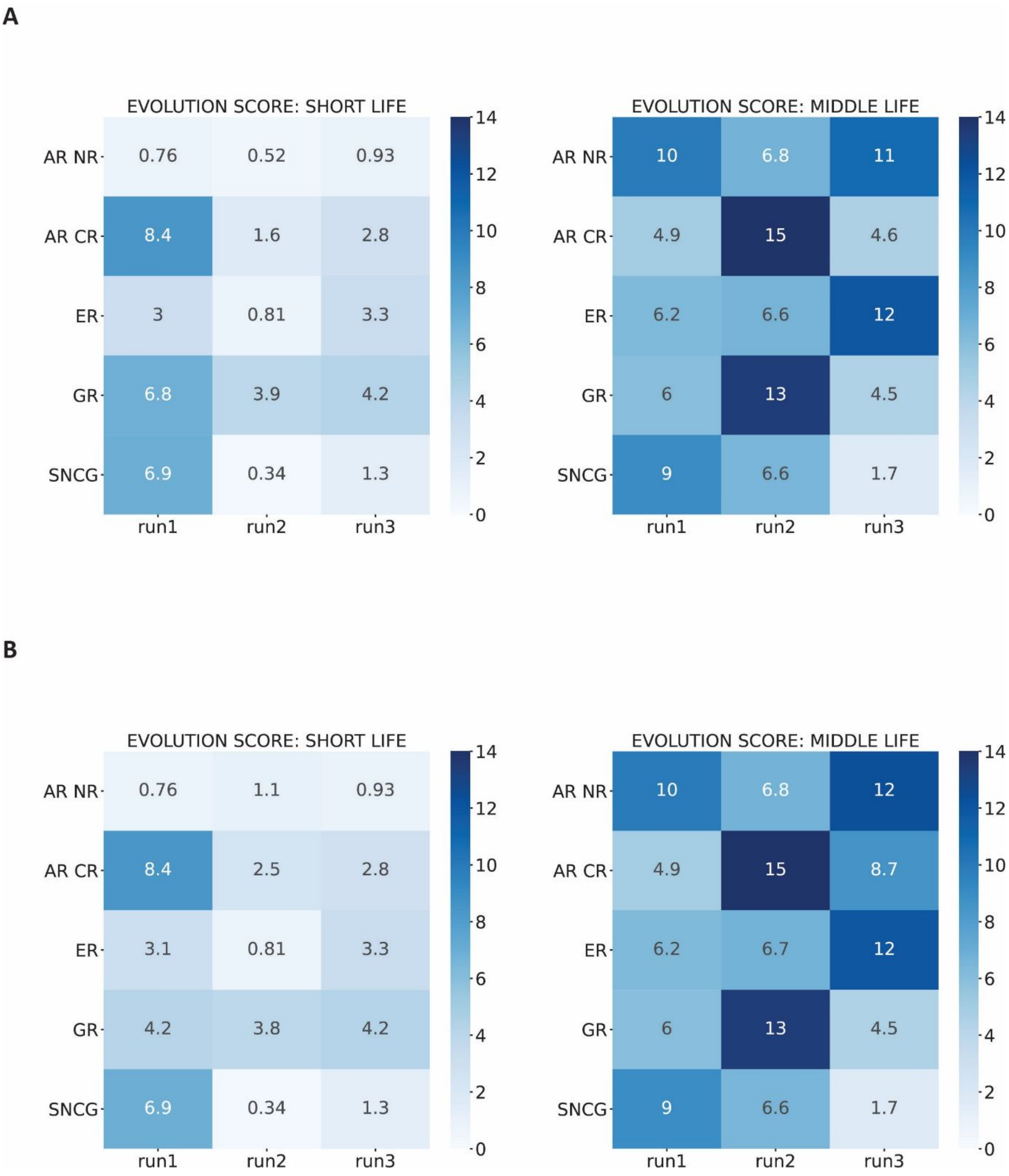

Figure S6. Evolution score results for different clustering parameters. A Evolution score calculated from clusters obtained by running the Gaussian Mixture clustering algorithm with the following parameters: n_init=100, tol=1e-4, max_iter=10000, reg_covar= 1e-4. **B** Evolution score calculated from clusters obtained by running the Gaussian Mixture clustering algorithm with the following parameters: n_init=100, tol=1e-4, max_iter=10000, reg_covar= 1e-5.

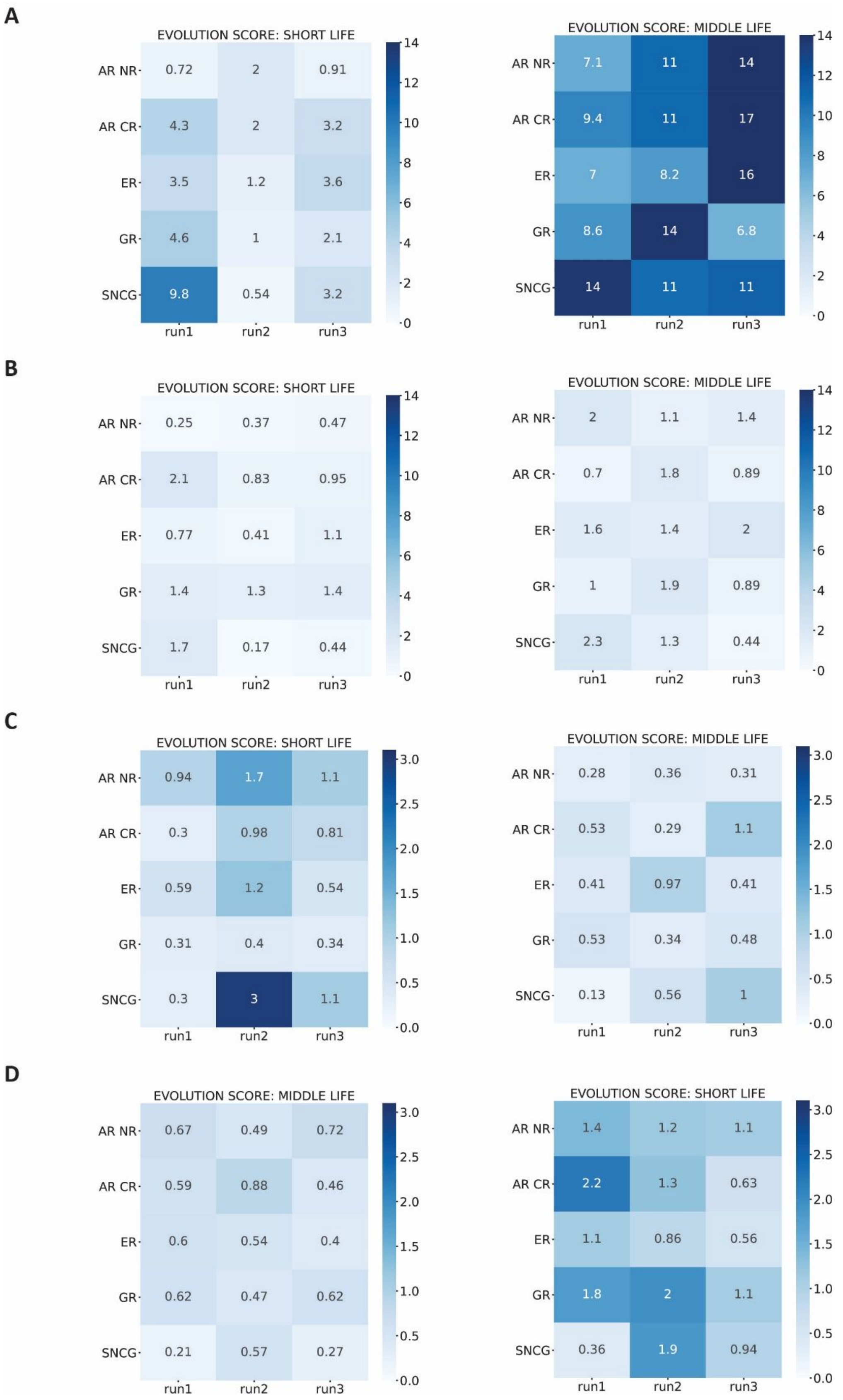

Figure S7. Evolution score results for different empirical definitions of evolution score. A Evolution score result calculated following the formulation:

$$E = \sum_{i=1}^{N-1} \frac{d_{i+1,i}}{s_i + s_{i+1}}$$

where s_i and s_{i+1} are the spread of cluster C_i and C_{i+1} respectively, $d_{i+1,i}$ is the 3D distance between the centroid of C_i and C_{i+1} and N is the total number of clusters. Here, distances between centroids and spread are calculated by using the total number of S, P and X contacts, without further normalization. **B** Evolution score result calculated following the formulation:

$$E = \frac{1}{N} \sum_{i=1}^{N-1} \frac{d_{i+1,i}}{s_i + s_{i+1}}$$

where s_i and s_{i+1} are the spread of cluster C_i and C_{i+1} respectively, $d_{i+1,i}$ is the 3D distance between the centroid of C_i and C_{i+1} and N is the total number of clusters. Here, distances between centroids and spread are calculated by using the number of P, S and X contacts divided by the total number of contacts in that specific configuration, in order to obtain their relative trends. **C** Evolution score result calculated following the formulation:

$$E = \frac{1}{N} \sum_{i=1}^{N-1} \frac{s_i + s_{i+1}}{d_{i+1,i}}$$

where s_i and s_{i+1} are the spread of cluster C_i and C_{i+1} respectively, $d_{i+1,i}$ is the 3D distance between the centroid of C_i and C_{i+1} and N is the total number of clusters. Here, distances between centroids and spread are calculated by using the total number of S, P and X contacts, without further normalization. **D** Evolution score result calculated following the formulation:

$$E = \frac{1}{\sqrt{N}} \left(\frac{1}{N} \sum_{i=1}^{N-1} \frac{s_i + s_{i+1}}{d_{i+1,i}} \right)$$

where s_i and s_{i+1} are the spread of cluster C_i and C_{i+1} respectively, $d_{i+1,i}$ is the 3D distance between the centroid of C_i and C_{i+1} and N is the total number of clusters. Here, distances between centroids and spread are calculated by using the number of P, S and X contacts divided by the total number of contacts in that specific configuration, in order to obtain their relative trends.

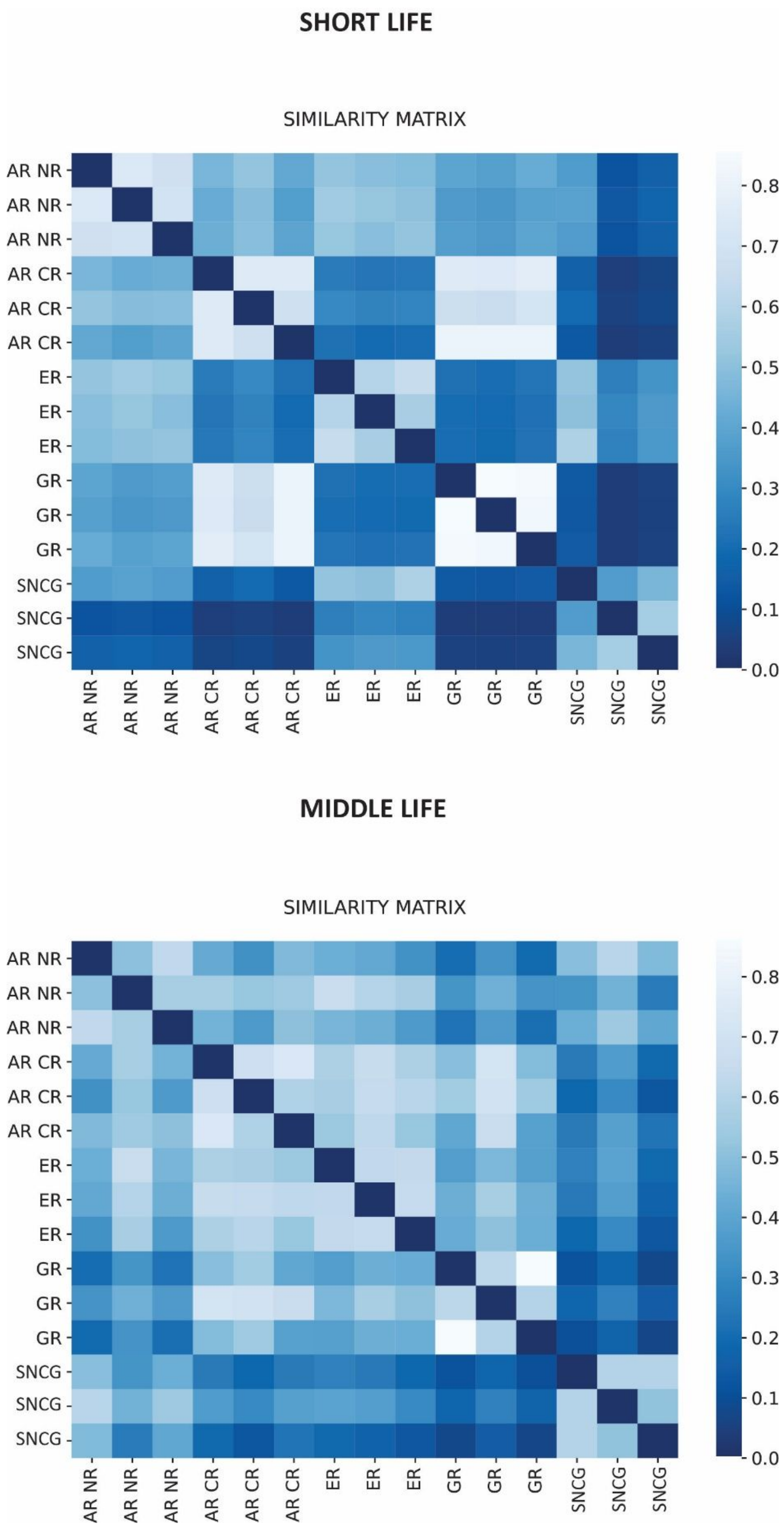

Figure S8. Similarity score for each pair of IDP/IDR in the dataset. Pairwise similarity scores for IDRs/IDPs. The scores were obtained by aligning strings corresponding to the topology reached by the protein in the centroid of the last occupied topological state during the MD run.

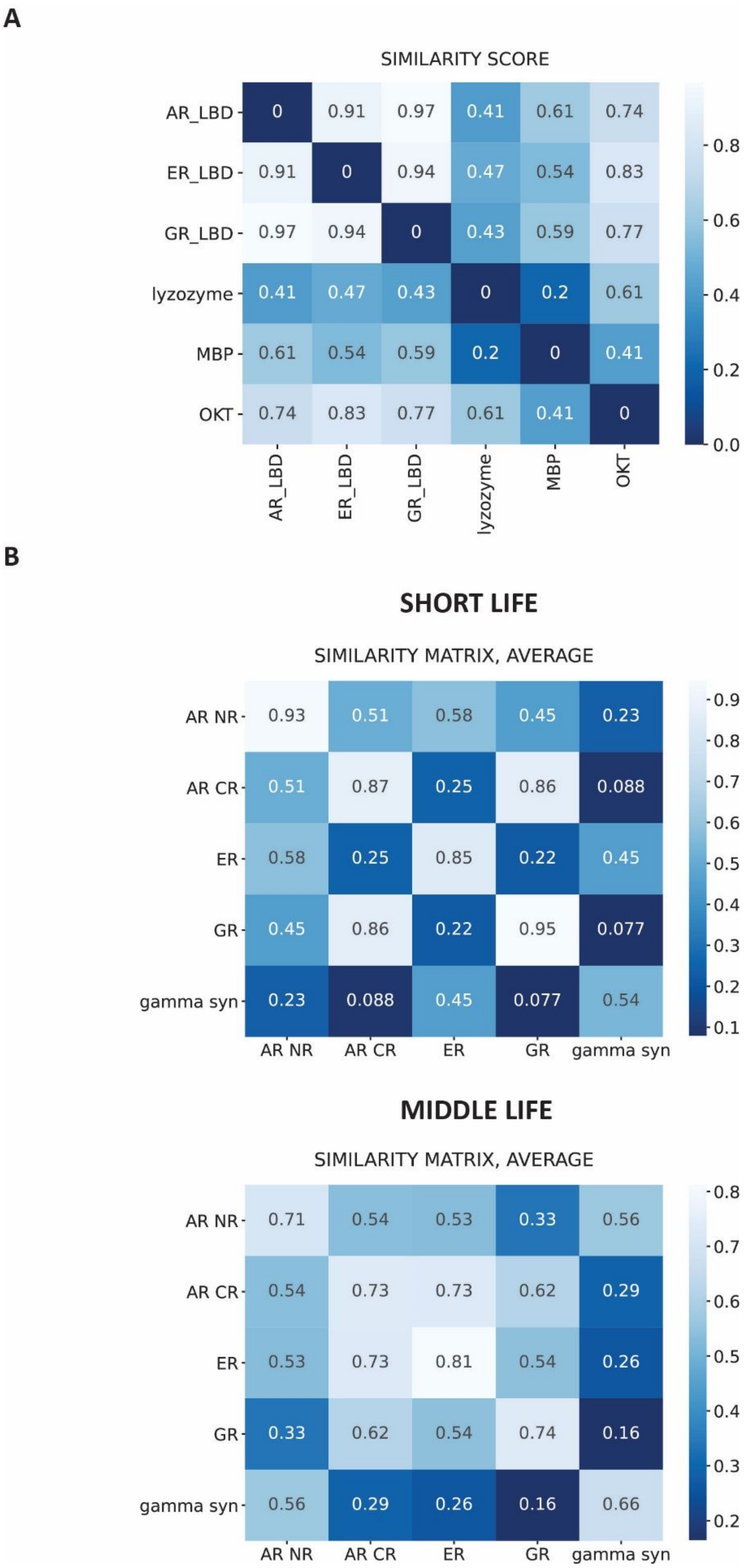

Figure S9. Similarity score as calculated by SequenceMatcher method. Pairwise similarity scores for IDRs/IDPs. The scores were obtained by running the SequenceMatcher.quick_ratio method on strings corresponding to the topology reached by the protein in the centroid of the last occupied topological state during the MD run. Scores obtained for all 3 MD runs are averaged into one value.

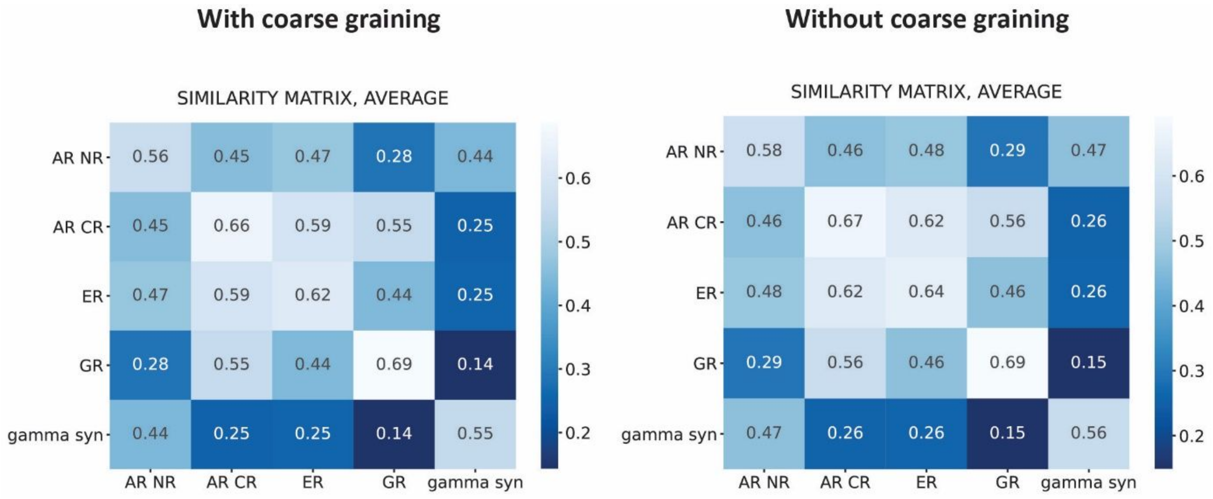

Figure S10. Similarity score (for middle life contacts) calculated with and without coarse graining of the topology strings. Coarse graining was performed by making substrings of 5 topological element. The value assigned to the substring is then the average of these 5 elements. We do not observe any significant difference in the patterns present in the similarity score matrix calculated with and without coarse graning, with differences in score amounting to a maximum of 0.03 for each element.

MIDDLE LIFE

Protein	Run 1	Run 2	Run 3
AR NR	5	7	9*
AR CR	6	8	7
ER	4	5	6
GR	6	7	5
SNCG	4	5	4

Table S1. Ideal number of clusters as identified by the BIC model, obtained by running the Gaussian Mixture clustering algorithm with the following parameters: n_init=100, tol=1e-4, max_iter=10000, reg_covar= 1e-4. In the case of AR NR run 3, the asterisk indicates a failure in the BIC model; the BIC score should decrease after we reach the ideal number of clusters, but in this case the score keeps rising indefinitely, even broadening the range of possible cluster numbers. Therefore, the number 9 was picked as the highest number of clusters which still provided good visual division into clusters of the data, without overfitting.

SHORT LIFE

Protein	Run 1	Run 2	Run 3
AR NR	3	2	2
AR CR	4	2	3
ER	4	2	3
GR	5	3	3
SNCG	4	2	3

Table S2. Ideal number of clusters as identified by the BIC model, obtained by running the Gaussian Mixture clustering algorithm with the following parameters: n_init=100, tol=1e-4, max_iter=10000, reg_covar= 1e-4.

MIDDLE LIFE

Protein	Run 1	Run 2	Run 3
AR NR	5	7	9*
AR CR	7	8	8
ER	4	5	6
GR	6	7	5
SNCG	4	5	4

Table S3. Ideal number of clusters as identified by the BIC model, obtained by running the Gaussian Mixture clustering algorithm with the following parameters: n_init=100, tol=1e-4, max_iter=10000, reg_covar= 1e-5. In the case of AR NR run 3, the asterisk indicates a failure in the BIC model; the BIC score should decrease after we reach the ideal number of clusters, but in this case the score keeps rising indefinitely, even broadening the range of possible cluster numbers. Therefore, the number 9 was picked as the highest number of clusters which still provided good visual division into clusters of the data, without overfitting.

SHORT LIFE

Protein	Run 1	Run 2	Run 3
AR NR	3	3	2
AR CR	4	3	3
ER	4	2	3
GR	4	3	3
SNCG	4	2	3

Table S4. Ideal number of clusters as identified by the BIC model, obtained by running the Gaussian Mixture clustering algorithm with the following parameters: n_init=100, tol=1e-4, max_iter=10000, reg_covar= 1e-5.

MIDDLE LIFE

Protein	Run 1	Run 2	Run 3
AR NR	5	7	9*
AR CR	7	8	8
ER	4	5	6
GR	6	7	5
SNCG	4	5	4

Table S5. Ideal number of clusters as identified by the BIC model, obtained by running the Gaussian Mixture clustering algorithm with the following parameters: n_init=100, tol=1e-4, max_iter=10000, reg_covar= 1e-6. In the case of AR NR run 3, the asterisk indicates a failure in the BIC model; the BIC score should decrease after we reach the ideal number of clusters, but in this case the score keeps rising indefinitely, even broadening the range of possible cluster numbers. Therefore, the number 9 was picked as the highest number of clusters which still provided good visual division into clusters of the data, without overfitting.

SHORT LIFE

Protein	Run 1	Run 2	Run 3
AR NR	3	3	2
AR CR	4	3	3
ER	4	2	3
GR	5	3	3
SNCG	4	2	3

Table S6. Ideal number of clusters as identified by the BIC model, obtained by running the Gaussian Mixture clustering algorithm with the following parameters: n_init=100, tol=1e-4, max_iter=10000, reg_covar= 1e-6.
